# Acupoint-to-Acupoint Penetrating Acupuncture as an Add-on to Conventional Care for Post-Stroke Spasticity: A Systematic Review and Meta-Analysis

**DOI:** 10.3390/healthcare14142163

**Published:** 2026-07-17

**Authors:** Mikyung Kim, Sul Gi Yoon, Chang-ho Han

**Affiliations:** 1 Department of Internal Medicine, Dongguk University Ilsan Oriental Hospital, Goyang-si 10326, Republic of Korea; 2 Department of Internal Medicine, Graduate School of Korean Medicine, Dongguk University, Seoul 04620, Republic of Korea; caramel.americano@gmail.com; 3 Department of Internal Medicine, College of Korean Medicine, Dongguk University WISE Campus, Gyeongju-si 38066, Republic of Korea; changho.han@gmail.com

**Keywords:** post-stroke spasticity, acupoint-to-acupoint penetrating acupuncture, acupuncture, complementary therapy, integrative medicine, stroke rehabilitation, systematic review, meta-analysis

## Abstract

**Highlights:**

**What are the main findings?**

- In 16 randomized controlled trials involving 1170 participants, acupoint-to-acupoint penetrating acupuncture (AAPA) used as an add-on to conventional post-stroke care was associated with reduced Modified Ashworth Scale scores compared with conventional care alone.
- Adjunctive AAPA was also associated with improvements in motor function, gait, balance, activities of daily living, and neurological deficits; however, the certainty of evidence was very low.

**What are the implications of the main findings?**

- AAPA may have possible add-on benefits as a complementary intervention for post-stroke spasticity, but the current evidence is insufficient to support definitive clinical recommendations.
- Further rigorous multicenter randomized trials in diverse rehabilitation settings are needed, with standardized spasticity outcomes, appropriate control conditions, blinded assessment where feasible, systematic safety monitoring, and longer follow-up.

**Abstract:**

**Background/Objectives**: Post-stroke spasticity is a common and disabling complication that impairs functional recovery. Acupuncture is used as a complementary intervention in stroke rehabilitation, but evidence for specific techniques, including acupoint-to-acupoint penetrating acupuncture (AAPA), remains limited. This study evaluated the adjunctive effects and safety of AAPA applied to limb acupoints in patients with post-stroke spasticity receiving conventional care. **Methods**: A systematic review and meta-analysis of randomized controlled trials was conducted. Nine databases and five clinical trial registries were searched from inception to June 2026. Trials compared AAPA plus conventional care with conventional care alone. The primary outcome was post-treatment spasticity severity. Risk of bias was assessed using RoB 2, and evidence certainty using GRADE. **Results**: Sixteen studies involving 1170 participants were included. Adjunctive AAPA significantly reduced Modified Ashworth Scale scores (SMD = −0.91, 95% CI: −1.23 to −0.59). Motor function, gait, balance, activities of daily living, and neurological deficits were also improved. However, most studies had a high overall risk of bias, no trial used a sham acupuncture comparator, and substantial heterogeneity was observed for several outcomes. The certainty of evidence for all GRADE-assessed clinical outcomes was very low. **Conclusions**: The findings suggest possible add-on benefits of AAPA when used with conventional post-stroke care, but the current evidence should be interpreted cautiously and is insufficient to support definitive clinical recommendations. Rigorous multicenter trials in diverse rehabilitation settings with standardized outcomes, systematic safety assessment, and longer follow-up are needed.

## 1. Introduction

Post-stroke spasticity is a common complication affecting approximately 25–40% of stroke survivors [1]. It is characterized by increased muscle tone and may be accompanied by impaired motor function, joint contracture, pain, and gait disturbance. These problems can restrict activities of daily living (ADL), reduce quality of life, limit rehabilitation outcomes, and increase caregiver burden and socioeconomic costs [2]. Current standard treatments, including oral antispastic medications, botulinum toxin injections, and physical and occupational therapy, have demonstrated clinical benefits. However, their use may be limited by adverse effects such as sedation or muscle weakness, the need for repeated injections, cost considerations, and restricted access to specialist rehabilitation services [2,3]. These limitations support the investigation of safe and accessible complementary approaches for managing post-stroke spasticity [3].

Acupuncture has been widely used as a complementary intervention for post-stroke spasticity, and several randomized controlled trials (RCTs) and systematic reviews have suggested potential benefits [4,5,6]. However, previous evidence syntheses have generally treated acupuncture as a broad and heterogeneous intervention, with limited attention to differences among specific needling techniques or stimulation methods [4,5,6]. Acupoint-to-acupoint penetrating acupuncture (AAPA) is a distinct acupuncture technique in which a single needle is inserted at one acupoint and advanced subcutaneously or intramuscularly toward another nearby acupoint without exiting the skin at the target point [7]. Compared with conventional acupuncture applied at individual acupoints, AAPA is intended to provide extended stimulation across a selected acupoint pathway. In clinical practice, it has been applied to paretic limbs in patients with motor deficits after stroke and is commonly combined with conventional rehabilitation therapy to support motor and functional recovery [7,8].

Despite its clinical use, the effectiveness and safety of AAPA as an adjunctive intervention for post-stroke spasticity have not been systematically established. A narrative review published in 2014 described its potential clinical value [7]. A subsequent systematic review addressing AAPA-related treatment for post-stroke foot inversion provided relevant preliminary evidence; however, its clinical scope was limited to foot inversion, and it did not isolate the adjunctive effect of AAPA from that of concomitant conventional therapies [9]. Therefore, the present systematic review and meta-analysis aimed to evaluate the adjunctive effects and safety of AAPA applied to spastic limbs in patients receiving conventional post-stroke care.

## 2. Materials and Methods

### 2.1. Study Design and Protocol Registration

This systematic review and meta-analysis of randomized controlled trials (RCTs) was conducted and reported in accordance with the Preferred Reporting Items for Systematic Reviews and Meta-Analyses (PRISMA) 2020 statement [10]. The completed PRISMA 2020 checklist is provided in Table A1 in Appendix A. The study protocol was prospectively registered in the International Prospective Register of Systematic Reviews (PROSPERO; registration number: CRD420261280992).

### 2.2. Data Sources and Search Strategy

We systematically searched nine electronic databases from their inception to 24 June 2026: PubMed, Embase, the Cochrane Library, the China National Knowledge Infrastructure (CNKI), Airiti Library, CiNii, ScienceON, Korean Studies Information Service System (KISS), and Oriental Medicine Advanced Searching Integrated System (OASIS). Clinical trial registries including ClinicalTrials.gov, the World Health Organization International Clinical Trials Registry Platform (WHO ICTRP), the Clinical Research Information Service (CRIS), the Chinese Clinical Trial Registry (ChiCTR), and the Japan Registry of Clinical Trials (jRCT) were also searched on 21 June 2026.

The search strategy combined controlled vocabulary terms, where applicable, and free-text terms related to “stroke”, “spasticity”, and “acupoint-to-acupoint penetrating acupuncture”. No database filters or search limits were applied for language, publication type, study design, or other bibliographic restrictions. The complete search strategies for all bibliographic databases and clinical trial registries, including the exact search terms, search fields, search dates, and filters or limits used, are presented in Table A2 in Appendix A to allow replication of the literature search. In addition, the reference lists of the included studies and relevant review articles were manually screened to identify potentially eligible studies.

### 2.3. Eligibility Criteria

Eligibility criteria were defined according to the population, intervention, comparator, outcome, and study design framework.

#### 2.3.1. Population

Studies involving patients with post-stroke spasticity were eligible. Studies were excluded if participants did not have a confirmed diagnosis of stroke or if spasticity was not identified as a clinical condition or treatment target.

#### 2.3.2. Intervention

Eligible studies evaluated acupoint-to-acupoint penetrating acupuncture (AAPA) applied to acupoints of the upper or lower limbs as an add-on intervention to conventional post-stroke care. AAPA was defined as a needling technique in which a needle is inserted at one acupoint and advanced subcutaneously or intramuscularly toward another acupoint without exiting the skin surface at the target point [7,8,11].

Trials were eligible only when the penetration route involved recognized meridian or extra acupoints. Studies were excluded if penetrating needling was applied exclusively to non-acupoint locations, including Ashi points, tender points, myofascial trigger points, muscular regions, anatomical landmarks, or investigator-defined needling sites. Studies that reported penetrating acupuncture without specifying the acupoints involved were also excluded.

To reduce clinical heterogeneity and focus on the local treatment of limb spasticity, only studies applying AAPA to limb acupoints were included. Studies were excluded if AAPA was applied to non-limb regions, such as the scalp, auricular region, or trunk, or if limb-based AAPA was not separable from other needling approaches.

#### 2.3.3. Comparator

Eligible trials were required to compare AAPA plus conventional care with the same conventional care without AAPA. Conventional care could include standard medical management, rehabilitation therapy, or other co-interventions administered equally in both groups.

Trials were excluded if the additional effect of AAPA could not be isolated, such as when the intervention group received AAPA together with another active treatment not provided to the control group. Trials directly comparing AAPA alone with another active treatment, without a common background treatment in both groups, were also excluded because they did not evaluate the adjunctive effect of AAPA.

#### 2.3.4. Outcomes

The primary outcome was the between-group difference in spasticity severity at the end of the predefined treatment period. Secondary outcomes included the total effective rate (TER), motor function, gait, balance, activities of daily living (ADL), neurological deficits, stroke-related quality of life, and adverse events (AEs). When TER was reported, it was included as a secondary outcome only if the definition of treatment response explicitly reflected improvement in spasticity. Studies were excluded if they did not report quantitative data for spasticity or clinically relevant outcomes related to spasticity.

#### 2.3.5. Study Design and Publication Type

Only RCTs were included. Studies without clearly described random allocation, non-randomized studies, observational studies, case reports, narrative reviews, systematic reviews, and study protocols without results were excluded. No restrictions were applied regarding publication status or publication type. In addition to peer-reviewed journal articles, gray literature sources, including theses, dissertations, conference abstracts, and trial registry records, were considered eligible if they met the predefined eligibility criteria and reported sufficient quantitative data on spasticity-related outcomes to permit effect estimation or inclusion in the evidence synthesis. Reports were excluded if they did not provide sufficient information to determine eligibility or did not present extractable outcome data relevant to post-stroke spasticity. Books were excluded.

### 2.4. Study Selection and Data Extraction

After duplicate records were removed, two reviewers independently screened the titles and abstracts of all identified records. Full-text articles of potentially eligible studies were retrieved and assessed independently according to the predefined eligibility criteria. Disagreements regarding study eligibility were resolved through discussion.

Two reviewers independently extracted data from the included studies using a predefined data extraction framework. The extracted information included study identification, publication type, sample size, participant characteristics, time since stroke onset, anatomical location of spasticity, intervention and comparator details, anatomical region treated with AAPA, treatment duration, number of treatment sessions, outcome assessment time points, and reported efficacy and safety outcomes.

To characterize the AAPA procedures, additional information was extracted regarding penetration routes, insertion and target acupoints, needle type and dimensions, insertion depth and angle, manual stimulation techniques, and needle retention time. The frequencies of individual acupoints and acupoint pairs used across studies were also recorded. For acupoint-pair frequency analyses, identical acupoint combinations applied in opposite directions were treated as distinct penetration routes.

For multi-arm studies, only data from the intervention and control groups that allowed evaluation of the adjunctive effect of AAPA were extracted.

### 2.5. Risk of Bias Assessment

Two reviewers independently assessed the risk of bias of the included RCTs using the Cochrane Risk of Bias 2 (RoB 2) tool [12]. The following five domains were evaluated: bias arising from the randomization process, bias due to deviations from intended interventions, bias due to missing outcome data, bias in measurement of the outcome, and bias in selection of the reported result.

Each domain was judged as having a low risk of bias, some concerns, or a high risk of bias. An overall risk-of-bias judgment was subsequently assigned for each study according to the RoB 2 guidance. Disagreements were resolved through discussion.

### 2.6. Data Synthesis and Statistical Analysis

Meta-analysis was performed when at least two studies reported sufficiently comparable data for the same outcome. When multiple assessment time points were reported, data obtained closest to the end of the planned treatment period were selected for analysis.

Statistical analyses were performed using Review Manager software (RevMan, version 5.4; Cochrane Collaboration, Copenhagen, Denmark). Because clinical and methodological heterogeneity was anticipated across studies, random-effects models were used for all pooled analyses.

For dichotomous outcomes, effect estimates were expressed as risk ratios (RRs) with 95% confidence intervals (CIs). For continuous outcomes, mean differences (MDs) with 95% CIs were calculated when the same measurement scale was used across studies. Standardized mean differences (SMDs) with 95% CIs were calculated when studies assessed the same clinical outcome using different scoring formats or measurement instruments.

Statistical heterogeneity was assessed using the Higgins’ I^2^ statistic. Subgroup analyses were conducted according to the anatomical location of spasticity when sufficient studies were available. Publication bias was planned to be assessed using funnel plots when at least 10 studies were available for a pooled outcome. Sensitivity analyses for the primary outcomes were performed using a leave-one-out approach to examine whether the pooled estimates were materially affected by any single study.

### 2.7. Certainty of Evidence

Two reviewers independently assessed the certainty of evidence for each meta-analyzed outcome using the Grading of Recommendations Assessment, Development and Evaluation (GRADE) approach [13]. The certainty of evidence was rated as high, moderate, low, or very low after considering risk of bias, inconsistency, indirectness, imprecision, and other relevant considerations. Disagreements were resolved through discussion.

## 3. Results

### 3.1. Study Selection

**Figure 1:**
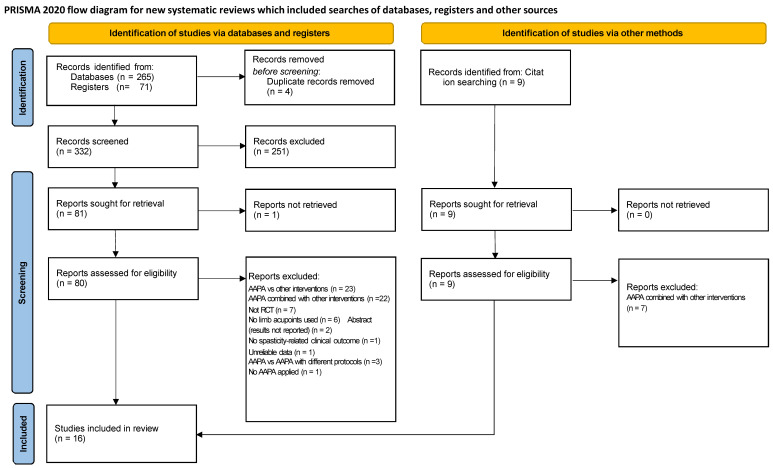
PRISMA flow diagram of study selection. AAPA, acupoint-to-acupoint penetrating acupuncture. RCT, randomized controlled trial.

The database search identified 265 records: 5 from PubMed, 13 from the Cochrane Library, 152 from CNKI, 48 from Airiti Library, 45 from CiNii, and 2 from ScienceOn. No records were retrieved from Embase, KISS, or OASIS. The clinical registry search identified 71 records (Table A2). After removing four duplicates, 332 records were screened by title and abstract, of which 251 were excluded. Full texts were sought for the remaining 81 records; one article could not be obtained, and 80 articles were assessed for eligibility. Bibliographic details of the full-text articles excluded after eligibility assessment, together with the reasons for exclusion, are provided in Table A3 in Appendix A. An additional nine full-text articles identified through manual reference screening were also assessed. Finally, 16 RCTs met the predefined eligibility criteria and were included in the systematic review and meta-analysis [14,15,16,17,18,19,20,21,22,23,24,25,26,27,28,29] (Figure 1).

### 3.2. Characteristics of the Included Studies

The characteristics of the included studies are summarized in Table 1.

#### 3.2.1. Study and Participant Characteristics

The 16 included RCTs were published between 2007 and 2026, and all were conducted in China and published in Chinese [14,15,16,17,18,19,20,21,22,23,24,25,26,27,28,29]. A total of 1170 participants were included, with study sample sizes ranging from 50 to 120 participants. The allocation ratio between the AAPA and control groups was approximately 1:1 in all studies.

The mean age of participants ranged from 51 to 66 years. The reported mean time since stroke onset ranged from 10 days to 105 days, except for one study that enrolled patients between 3 months and 3 years after stroke [15]. The targeted anatomical locations of spasticity were the ankle in five studies [15,16,17,18,21], the upper limb in four studies [22,23,25,28], the hand in two studies [19,20], the lower limb in one study [29], and the limbs without further anatomical specification in four studies [14,24,26,27].

#### 3.2.2. Concomitant and Control Interventions

All included studies evaluated AAPA as an add-on intervention to background conventional care. Most studies provided rehabilitation therapy in both groups [14,15,16,17,18,19,20,21,22,24,25,26,27,28,29]. In some trials, additional treatments were delivered equally as part of background care, including body acupuncture [15,19,29], scalp acupuncture [15], electroacupuncture [27], or herbal medicine [24]. In all studies, the control group received the same background care without the addition of AAPA. None of the included trials used sham acupuncture or a waitlist control.

#### 3.2.3. Characteristics of AAPA Interventions

##### Treatment Region, Duration, and Number of Sessions

All included studies applied AAPA to limb acupoints corresponding to the anatomical region affected by spasticity. Studies targeting ankle spasticity used lower-limb acupoints [15,16,17,18,21], studies targeting hand spasticity used upper-limb acupoints [19,20], and studies targeting upper-limb or lower-limb spasticity applied AAPA to acupoints in the affected limb regions [14,22,23,24,25,26,27,28,29].

Most studies applied AAPA to the affected side. Four studies did not clearly report the side of treatment [16,19,29], and no study explicitly reported contralateral or bilateral application.

Treatment duration ranged from 2 to 12 weeks, with 4 weeks being the most frequently used duration in ten studies [15,16,18,19,20,21,22,27,28,29]. The mean treatment duration was 5.0 ± 2.6 weeks. The number of treatment sessions ranged from 12 to 60, with a mean of 28.1 ± 13.6 sessions. In studies using a 4-week intervention period, treatment was generally delivered six or seven times per week.

##### Acupoint Selection Patterns

Detailed procedural characteristics of AAPA are presented in Table A4 in Appendix A. The number of acupoint pairs used in individual studies ranged from 1 to 10, with a mean of 4.8 ± 2.7 pairs per study.

Across the included studies, 36 distinct directional acupoint pairs were identified. The most frequently used directional pair was GB34 → SP9, which was identified in nine studies. This was followed by LI4 → SI3 and GB40 → KI6. Among the ten most frequently used penetration routes, upper- and lower-limb acupoint pairs were equally represented, with five routes identified for each anatomical region (Table 2A).

Across the included studies, 45 individual acupoints were identified. GB34 was the most frequently used acupoint, appearing in 12 studies, followed by SP9 and LI4, which appeared in 11 and 10 studies, respectively (Table 2B).

##### Technical Characteristics of Needling

The technical characteristics of AAPA needling are summarized in Table A4 in Appendix A. Filiform needles were the most commonly used needle type [14,16,17,18,19,20,21,23,29]. Silver needles were used in two studies [22,27], and long needles were used in two studies [24,28]. Needle diameter ranged from 0.25 to 0.60 mm, and needle length ranged from 25 to 100 mm. The most commonly reported needle diameter and length were 0.30 mm and 40 mm, respectively.

Insertion angle was not reported in most studies. When described, oblique, perpendicular, or transverse insertion was selected according to the acupoint pair and anatomical region. Ten studies reported manual stimulation techniques after insertion, including twirling and/or lifting–thrusting [14,15,16,17,18,19,20,22,24,29]. Needle retention time was consistently reported as 20–30 min.

#### 3.2.4. Outcome Measures

All included studies assessed outcomes immediately after completion of the planned intervention period. Only one study reported an interim outcome assessment [21], and none reported post-treatment follow-up outcomes.

Ten studies reported Modified Ashworth Scale (MAS) outcomes [15,19,20,22,23,25,26,27,28,29], and one study assessed spasticity using the Composite Spasticity Index [18]. Nine studies assessed spasticity-related treatment response using the total effective rate (TER) [15,16,17,19,21,22,26,27,28].

Motor function was most commonly evaluated using the Fugl–Meyer Assessment (FMA) [14,15,16,17,18,19,20,21,22,23,24,25,26,27,28,29]. Two studies reported gait parameters [21,26], two reported balance using the Berg Balance Scale (BBS) [24,27], and seven reported activities of daily living using the Modified Barthel Index (MBI) [14,18,20,22,23,26,28]. Three studies evaluated neurological deficits using the National Institutes of Health Stroke Scale (NIHSS) [22,25,26], one assessed stroke-related quality of life [28], and three reported adverse events (AEs) [23,26,28].

### 3.3. Risk of Bias Assessment

**Figure 2:**
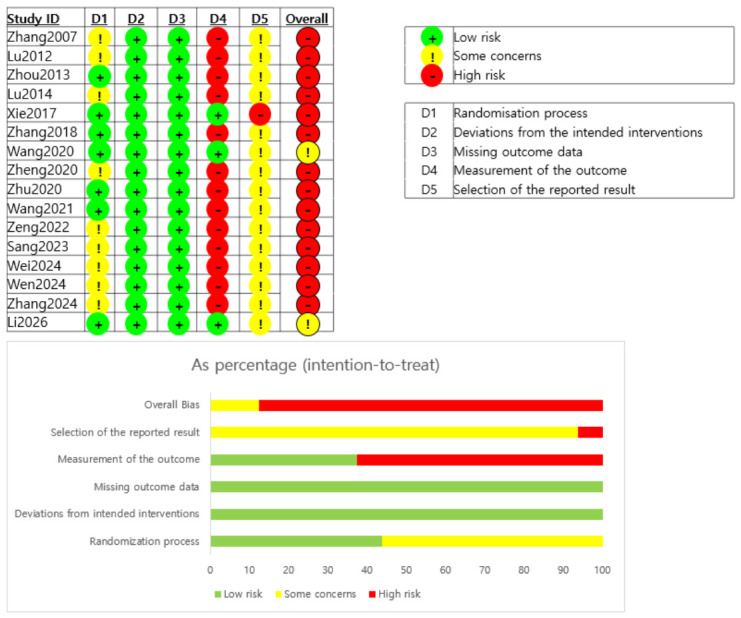
Risk of bias assessment [14,15,16,17,18,19,20,21,22,23,24,25,26,27,28,29].

The results of the RoB 2 assessment are shown in Figure 2. For bias arising from the randomization process, seven studies were judged to have a low risk of bias because they explicitly reported adequate random sequence generation and allocation concealment [16,18,19,20,22,23,29]. The remaining studies were judged as having some concerns because reporting of the randomization process was insufficient.

For bias due to deviations from intended interventions, all studies were judged to have a low risk of bias. Although participant and practitioner blinding was not reported, this was considered unlikely to have resulted in deviations from the assigned intervention.

For bias due to missing outcome data, all studies were judged to have a low risk of bias because the number of participants analyzed was comparable to the number randomized.

For bias in measurement of the outcome, only three studies explicitly reported assessor blinding [18,20,29]. The remaining studies were judged to have a high risk of bias because outcome assessment may have been influenced by knowledge of the intervention assignment.

For bias in selection of the reported result, most studies were judged as having some concerns because no prespecified protocol or trial registration record was available. One study was judged to have a high risk of bias in this domain because discrepancies were identified between the reported outcomes and those described in its protocol publication [18].

Overall, two studies were judged as having some concerns [20,29], whereas the remaining 14 studies were judged as having a high overall risk of bias.

### 3.4. Effects of Adjunctive AAPA

#### 3.4.1. Spasticity

**Figure 3:**
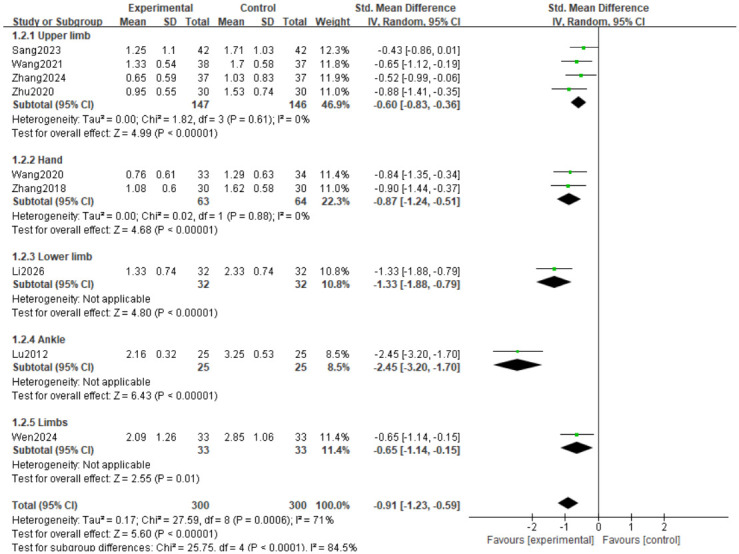
Forest plot of adjunctive effects of acupoint-to-acupoint penetrating acupuncture on Modified Ashworth Scale (MAS) scores [15,19,20,22,23,25,27,28,29].

Of the ten studies reporting MAS outcomes [15,19,20,22,23,25,26,27,28,29], nine studies involving 600 participants provided data suitable for meta-analysis [15,19,20,22,23,25,27,28,29]. Because MAS outcomes were reported using different formats, SMDs were calculated. Adjunctive AAPA significantly reduced MAS scores compared with conventional care alone (SMD = −0.91, 95% CI: −1.23 to −0.59, I^2^ = 71%) (Figure 3). Subgroup analyses by anatomical location showed a consistent direction of effect favoring AAPA across all subgroups (Figure 3). Within-subgroup statistical heterogeneity was reduced or not estimable. The certainty of evidence for MAS was rated as very low because most contributing studies had a high risk of bias and substantial statistical heterogeneity was observed in the overall analysis (Table 3).

**Figure A1:**
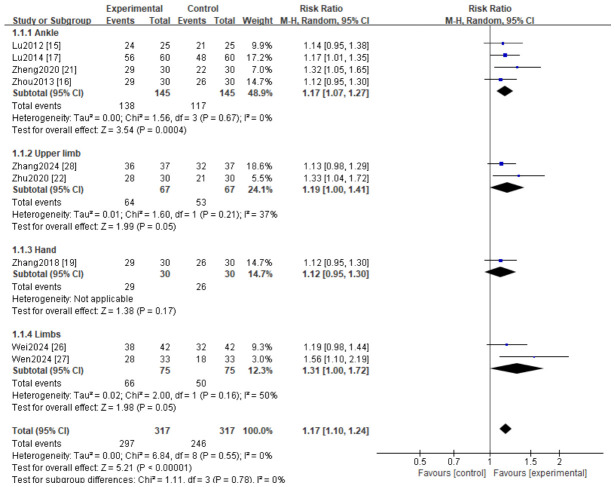
Forest plot of adjunctive effects of acupoint-to-acupoint penetrating acupuncture on spasticity-related treatment response (total effective rate).

As a secondary supportive response outcome, TER was analyzed only in nine studies in which the response definition explicitly reflected improvement in spasticity [15,16,17,19,21,22,26,27,28]. Adjunctive AAPA was associated with a higher TER than conventional care alone (RR = 1.17, 95% CI: 1.10–1.24; I^2^ = 0%) (Figure A1 in Appendix A).

#### 3.4.2. Motor Function

**Figure 4:**
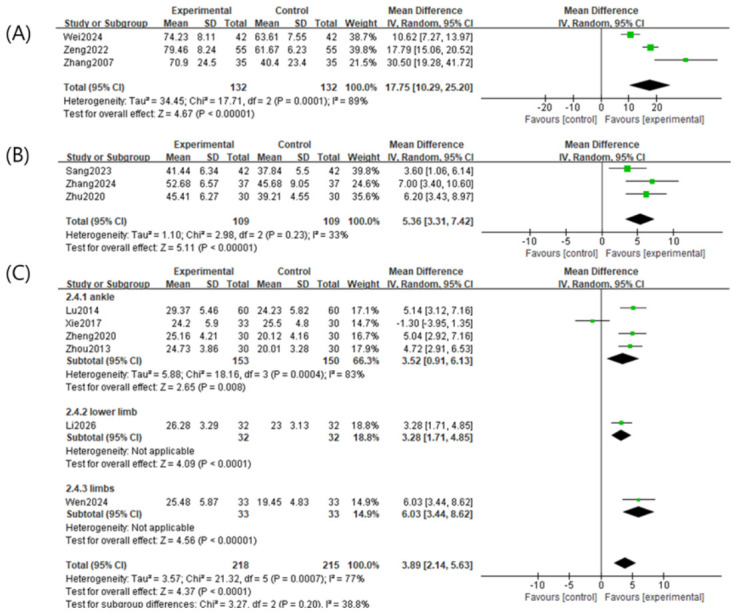
Forest plot of adjunctive effects of acupoint-to-acupoint penetrating acupuncture on motor function (Fugl–Meyer Assessment): (**A**) motor component (100 points) [14,24,26]; (**B**) upper limb (66 points) [22,25,28]; and (**C**) lower limb (34 points) [16,17,18,21,27,29].

Three RCTs involving 264 participants reported total FMA motor scores and were included in the pooled analysis [14,24,26]. Adjunctive AAPA significantly improved total FMA motor scores compared with conventional care alone (MD = 17.75, 95% CI: 10.29–25.20; I^2^ = 89%) (Figure 4A).

Three RCTs involving 218 participants reported upper-limb FMA subscores [22,25,28]. Adjunctive AAPA significantly improved upper-limb motor function (MD = 5.36, 95% CI: 3.31–7.42; I^2^ = 33%) (Figure 4B).

Six RCTs involving 433 participants reported lower-limb FMA subscores [16,17,18,21,24,29]. Adjunctive AAPA significantly improved lower-limb motor function (MD = 3.89, 95% CI: 2.14–5.63; I^2^ = 77%) (Figure 4C). Subgroup analyses according to spasticity location showed a consistent direction of effect favoring AAPA across ankle spasticity, lower-limb spasticity, and limb spasticity without further anatomical specification.

The certainty of evidence for motor outcomes was rated as very low. Downgrading was mainly attributable to concerns regarding risk of bias, inconsistency, and/or imprecision, depending on the outcomes (Table 3).

#### 3.4.3. Gait

**Figure 5:**
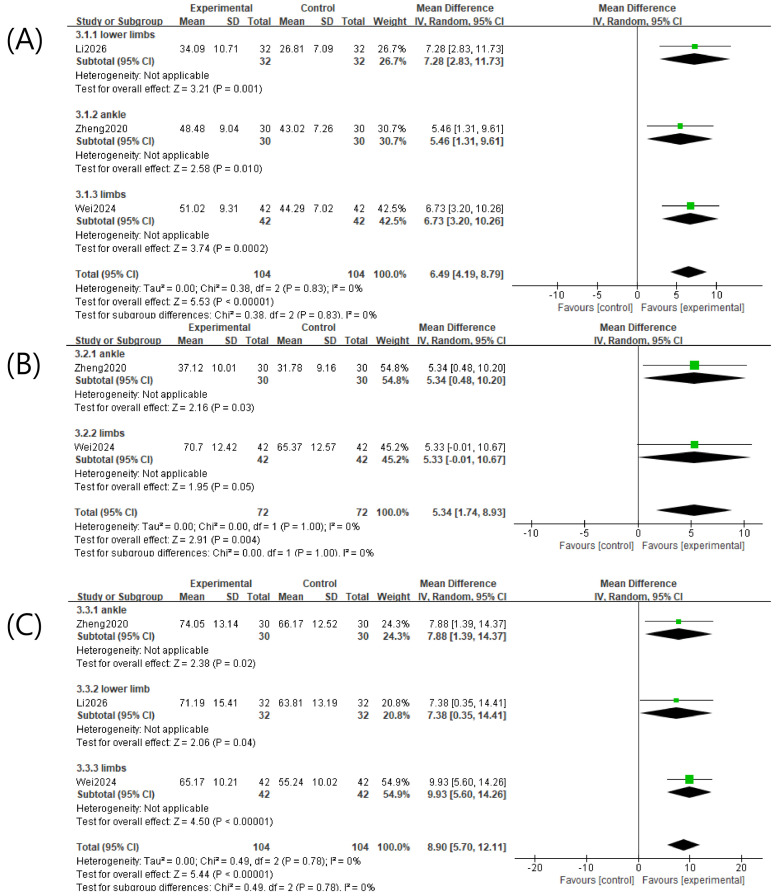
Forest plot of adjunctive effects of acupoint-to-acupoint penetrating acupuncture on gait parameters: (**A**) gait speed (cm/s) [21,26,29]; (**B**) step length of affected side (cm) [21,26]; and (**C**) cadence (step/minute) [21,26,29].

Three RCTs involving 208 participants reported gait speed [21,26,29]. Adjunctive AAPA significantly improved gait speed compared with conventional care alone (MD = 6.49 cm/s, 95% CI: 4.19–8.79; I^2^ = 0%) (Figure 5A).

Two RCTs involving 144 participants reported step length of the affected side [21,26]. Adjunctive AAPA significantly increased step length compared with conventional care alone (MD = 5.34 cm, 95% CI: 1.74–8.93; I^2^ = 0%) (Figure 5B).

Three RCTs involving 208 participants reported cadence [21,26,29]. Adjunctive AAPA significantly improved cadence compared with conventional care alone (MD = 8.90 steps/min, 95% CI: 5.70–12.11; I^2^ = 0%) (Figure 5C). Subgroup analysis by spasticity location showed a consistent direction of effect favoring AAPA, with no observed statistical heterogeneity within the pooled gait outcomes.

The certainty of evidence for gait outcomes was rated as very low. Gait speed, step length, and cadence were downgraded because of very serious risk of bias and serious imprecision (Table 3).

#### 3.4.4. Balance

**Figure 6:**
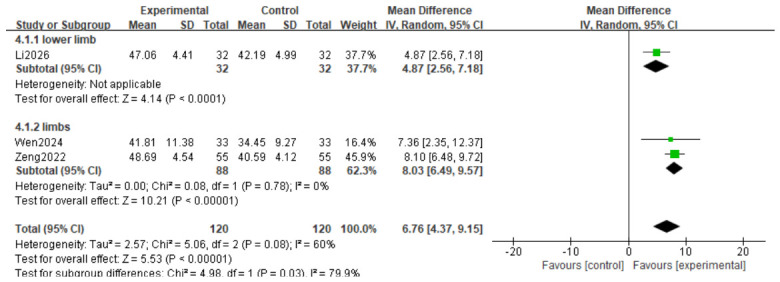
Forest plot of adjunctive effects of acupoint-to-acupoint penetrating acupuncture on balance (Berg Balance Scale) [24,27,29].

Three RCTs involving 240 participants reported BBS scores [24,27,29]. Adjunctive AAPA significantly improved balance compared with conventional care alone (MD = 6.76, 95% CI: 4.37–9.15; I^2^ = 60%) (Figure 6). In subgroup analysis by spasticity location, the direction of effect consistently favored AAPA, and within-subgroup heterogeneity was lower or not estimable. The certainty of evidence for balance function was rated as very low because of very serious risk of bias and serious imprecision (Table 3).

#### 3.4.5. Activities of Daily Living

**Figure 7:**
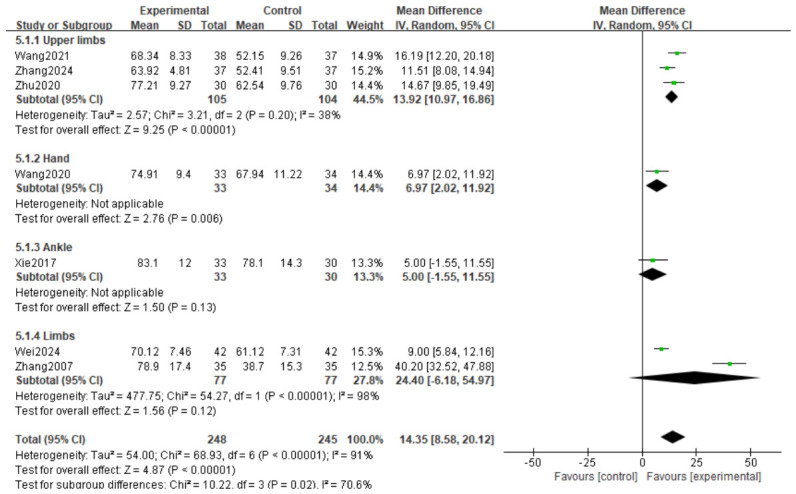
Forest plot of adjunctive effects of acupoint-to-acupoint penetrating acupuncture on activities of daily living (Modified Barthel Index) [14,18,20,22,23,26,28].

Seven RCTs involving 493 participants reported MBI scores and were included in the meta-analysis [14,18,20,22,23,26,28]. Adjunctive AAPA significantly improved ADL performance compared with conventional care alone (MD = 14.33, 95% CI: 8.58–20.12; I^2^ = 91%) (Figure 7). Subgroup analyses by spasticity location showed generally similar directions of effect, although statistical significance and heterogeneity varied across subgroups. The certainty of evidence for ADL performance was rated as very low, mainly because of concerns regarding risk of bias and inconsistency (Table 3).

#### 3.4.6. Neurological Deficits

**Figure 8:**
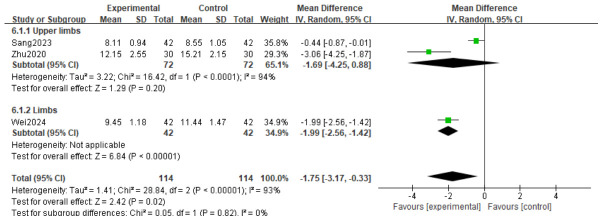
Forest plot of adjunctive effects of acupoint-to-acupoint penetrating acupuncture on neurological deficits (National Institutes of Health Stroke Scale) [22,25,26].

Three RCTs involving 228 participants reported NIHSS scores [22,25,26]. Adjunctive AAPA significantly reduced neurological deficit scores compared with conventional care alone (MD = −1.75, 95% CI: −3.17 to −0.33; I^2^ = 93%) (Figure 8). In the subgroup analysis of studies involving upper-limb spasticity, the direction of effect favored AAPA, but the pooled estimate was not statistically significant. The certainty of evidence for neurological deficits was rated as very low, mainly because of concerns regarding risk of bias, indirectness, and imprecision (Table 3).

### 3.5. Publication Bias and Sensitivity Analysis

Publication bias was not assessed because fewer than 10 studies were included in each meta-analysis. Leave-one-out sensitivity analyses were performed for the primary spasticity outcomes. Sequential exclusion of individual studies did not materially change the direction or statistical significance of the pooled effects, indicating that the primary findings were not driven by a single study (Table A5 in Appendix A).

### 3.6. Safety

Four RCTs involving 297 participants reported AEs [23,26,28,29]. Three studies involving 223 participants explicitly reported that no AEs occurred during the study period [23,26,29]. One study reported one case of localized subcutaneous hematoma among 37 participants in the AAPA group [28]. No serious AEs were reported in any study. Because safety outcomes were reported in only four studies and the number of events was very small, a meta-analysis of AEs was not performed.

### 3.7. Certainty of Evidence

The GRADE assessment is summarized in Table 3. The certainty of evidence was rated as very low for all meta-analyzed outcomes included in the summary of findings. Downgrading was mainly attributable to concerns regarding risk of bias, inconsistency, imprecision, and, for NIHSS, indirectness. Overall, the observed benefits of adjunctive AAPA were supported by evidence of very low certainty.

## 4. Discussion

### 4.1. Summary of Main Findings

This systematic review and meta-analysis suggests that acupoint-to-acupoint penetrating acupuncture (AAPA), when added to conventional post-stroke care, may provide additional benefits for patients with post-stroke limb spasticity. The most directly relevant pooled estimate for spasticity severity showed that adjunctive AAPA reduced Modified Ashworth Scale (MAS) scores (SMD = −0.91). Leave-one-out sensitivity analyses did not materially alter the direction or statistical significance of these findings, suggesting that the pooled estimates for the primary outcomes were not driven by any single study.

However, these statistically significant pooled effects should be interpreted cautiously rather than as confirmatory evidence. Fourteen of the 16 included studies were judged to have a high overall risk of bias, and the certainty of evidence for all GRADE-assessed clinical outcomes was very low. In addition, none of the included trials used a sham acupuncture comparator, and substantial heterogeneity was observed for several outcomes. Therefore, the observed effect estimates may overstate the true adjunctive effect of AAPA and should be interpreted within these important epistemic limitations.

A previous meta-analysis evaluating acupuncture more broadly for post-stroke spasticity reported beneficial effects on total effective rate (TER) in both upper- and lower-limb spasticity and on MAS scores [4]. The magnitude and direction of the effects observed in the present review were broadly comparable to those previous findings. However, such comparisons are indirect because the previous review evaluated heterogeneous acupuncture interventions, whereas the present review specifically focused on limb-based AAPA administered as an add-on to conventional care. Therefore, the current findings should not be interpreted as demonstrating the superiority of AAPA over other acupuncture modalities. Rather, they provide technique-specific evidence suggesting that AAPA warrants further evaluation as a complementary intervention for post-stroke spasticity.

Spasticity may adversely affect motor function, gait, balance, and independence in activities of daily living (ADL). In the present review, adjunctive AAPA was associated with statistically significant improvements in motor function, gait parameters, balance, ADL, and neurological deficits. The observed mean differences in upper-limb Fugl–Meyer Assessment (FMA) scores (MD = 5.36), lower-limb FMA scores (MD = 3.89), Modified Barthel Index (MBI) scores (MD = 14.33), and Berg Balance Scale (BBS) scores (MD = 6.76) exceeded previously reported minimal clinically important difference (MCID) thresholds in stroke populations [30,31,32]. These comparisons suggest that some functional improvements may be clinically relevant in addition to being statistically significant. Nevertheless, the MCID thresholds were derived from different clinical contexts, and the certainty of evidence for these functional outcomes was very low. Accordingly, the clinical importance of the observed effects remains uncertain and should be confirmed in rigorously designed trials.

Several included studies [15,16,17,18,21] targeted ankle spasticity, which is clinically related to equinus or equinovarus patterns involving muscles such as the gastrocnemius, soleus, and tibialis posterior [33]. Because botulinum toxin type A injection is an established focal treatment for post-stroke spasticity [34], the present findings should not be interpreted as suggesting that AAPA can replace standard focal treatments. Rather, AAPA may be considered a potential complementary add-on to conventional rehabilitation and focal spasticity management, a possibility that should be tested in pragmatic trials with clear documentation of concomitant treatments and rehabilitation intensity.

### 4.2. Mechanistic Considerations

Previous studies have proposed several mechanisms through which acupuncture may influence post-stroke spasticity, including modulation of spinal reflex excitability, reduction of motor neuron hyperexcitability, changes in neurotransmitter activity, and enhancement of ipsilesional motor cortex activation [4,35,36]. However, mechanistic evidence specifically addressing AAPA remains limited.

Compared with acupuncture applied separately at individual acupoints, AAPA uses a single needle advanced between two acupoints and may therefore provide a different pattern or extent of peripheral stimulation. Preliminary acupuncture-related evidence has suggested possible associations between treatment and changes in excitatory or inhibitory amino-acid neurotransmission, including glutamate and γ-aminobutyric acid activity [37]. However, the available evidence does not establish whether AAPA has mechanisms distinct from those of other acupuncture techniques. It also remains unclear whether the observed adjunctive effects are attributable to an expanded stimulation area, altered afferent sensory input, the specific penetration route, or contextual effects arising from its use alongside conventional rehabilitation. Future studies incorporating electrophysiological, neuroimaging, or biomarker-based outcomes are required to investigate these hypotheses.

### 4.3. Acupoint Selection Patterns

The acupoints and acupoint pairs frequently identified in this review largely overlapped with those commonly selected in conventional acupuncture for post-stroke spasticity [4]. This finding suggests that AAPA does not necessarily employ a fundamentally different acupoint selection framework; rather, it may represent a modification of the stimulation technique applied within established acupuncture treatment strategies.

A comparison with our previous systematic review of AAPA for post-stroke dysphagia showed substantially different commonly used acupoint pair combinations [38]. This descriptive difference suggests that acupoint selection within the AAPA framework may be adapted to the clinical manifestation being treated. However, the present review was not designed to determine whether particular acupoint pairs or penetration routes are associated with greater efficacy. Future trials should report AAPA procedures in sufficient detail and evaluate whether standardized or symptom-specific protocols influence treatment outcomes.

### 4.4. Safety

Among the 297 participants across the four RCTs reporting adverse events (AEs), only one case of localized subcutaneous hematoma was documented [23,26,28,29]. No serious AEs were reported. Although these findings do not indicate a major safety concern, safety outcomes were reported in only a small proportion of the included studies, and the number of observed events was very limited. Therefore, no definitive conclusions can be drawn regarding the safety of AAPA. Future clinical trials should systematically monitor and report AEs, particularly because AAPA may involve longer needles and deeper or more extensive tissue penetration than conventional acupuncture at individual acupoints.

### 4.5. Limitations

Several limitations should be considered when interpreting the findings of this review. First, the methodological quality of the included evidence was limited. Fourteen of the 16 included studies were judged to have a high overall risk of bias using the Cochrane RoB 2 tool, and the certainty of evidence for all GRADE-assessed clinical outcomes was very low. Assessor blinding was rarely reported, and no trial used a sham acupuncture comparator. Because all included studies evaluated AAPA as an add-on to background conventional care, the current evidence cannot determine whether the observed benefits were attributable to the specific physiological effects of AAPA, nonspecific needling effects, contextual effects, or interactions with concomitant rehabilitation. These limitations reduce confidence in the pooled estimates and suggest that the observed effects may have been overestimated.

Second, substantial clinical and statistical heterogeneity was observed, which limits the precision and applicability of the pooled estimates. The included studies differed in anatomical location of spasticity, time since stroke onset, baseline spasticity severity, stroke-related functional status, background rehabilitation, concomitant treatments, treatment frequency and duration, needling procedures, and AAPA penetration routes. Heterogeneity remained for several outcomes, including MAS, total and lower-limb FMA scores, MBI scores and NIHSS scores, even after subgroup analysis according to the anatomical location of spasticity. Although random-effects models, anatomical-location subgroup analyses, leave-one-out sensitivity analyses, and GRADE inconsistency assessments were used to address this heterogeneity, these approaches could not fully resolve the underlying clinical diversity. Therefore, the pooled estimates should be understood as broad average estimates of the adjunctive effect of limb-based AAPA across clinically diverse populations and treatment contexts, rather than as evidence for a uniform effect across specific stroke subtypes, spasticity severities, time-since-stroke categories, anatomical phenotypes, or AAPA protocols. Because only a limited number of studies were available for individual outcomes, meta-regression or more detailed exploratory analyses could not be performed.

Third, concerns remain regarding the validity and consistency of the outcome measures used to assess spasticity. TER, *a* commonly reported outcome in the included studies, is not an internationally standardized measure of spasticity. Moreover, in several included trials, the definition of clinical response extended beyond a reduction in spasticity and incorporated improvements in neurological deficits or functional recovery. This variability in response criteria introduced conceptual ambiguity and limited the interpretability of the pooled TER estimate. Although the MAS is widely used in clinical practice and research, it is susceptible to examiner-dependent variability and subjective judgment [39]. Because spasticity is associated with altered reflex excitability, the inclusion of objective and quantitative measures, such as electrophysiological parameters including the H-reflex, could strengthen the validity of future evaluations [40]. None of the included studies incorporated objective measures of spasticity.

Fourth, all included studies were conducted in China and published in Chinese, and most had relatively small sample sizes. Because acupuncture trials from certain countries have been reported to show a high proportion of favorable findings, positive outcome or publication bias may have inflated the pooled estimates [41]. The external applicability of the findings is also uncertain, as the intensity of background rehabilitation, access to physiotherapy or occupational therapy, use of orthoses, oral antispastic medications, and focal treatments such as botulinum toxin injection may differ substantially across healthcare systems [34]. Accordingly, the observed adjunctive effects of AAPA may not be directly generalizable to Western or other non-Chinese rehabilitation contexts. In addition, although we made extensive efforts to identify the available evidence relevant to this topic, the possibility of missing eligible studies cannot be completely excluded.

Fifth, this review included only limb-based AAPA interventions. This decision was made to improve clinical comparability and focus on the local treatment of limb spasticity. However, it limits the generalizability of the findings to other penetrating acupuncture approaches involving the scalp, trunk, or distal acupoint strategies.

Finally, safety and long-term outcomes were insufficiently assessed. Adverse events were reported in only three studies, and all included studies assessed outcomes at or near the completion of treatment, without post-treatment follow-up. Therefore, no definitive conclusions can be drawn regarding the safety or durability of AAPA. Future studies should include adequately powered, multicenter RCTs conducted in diverse clinical settings, use standardized and objective spasticity outcomes, incorporate blinded outcome assessment and appropriate control conditions where feasible, systematically monitor adverse events, clearly document concomitant rehabilitation and spasticity management, and include longer-term follow-up.

## 5. Conclusions

This systematic review and meta-analysis suggests that acupoint-to-acupoint penetrating acupuncture (AAPA) applied to limb acupoints as a complementary add-on to conventional post-stroke care may reduce spasticity and improve related functional outcomes. However, the certainty of the available evidence was very low. The findings were limited by the high overall risk of bias in most included studies, substantial heterogeneity among studies, the absence of sham-controlled trials, possible publication or positive outcome bias, and limited safety and follow-up data. Therefore, the current evidence should be interpreted cautiously and is insufficient to support definitive clinical recommendations. Future well-designed, adequately powered, multicenter randomized controlled trials are needed in diverse rehabilitation settings, with standardized and objective spasticity outcomes, blinded outcome assessment where feasible, appropriate control conditions, clear reporting of concomitant rehabilitation and spasticity management, systematic monitoring of adverse events, and longer-term follow-up.

## Figures and Tables

**Table 1:** Characteristics of included studies.

Study ID	Sample Size (M/F)	Age (yr) *	TSS *	SP Location	Interventions	Tx Duration (wk)	Session No.	Outcome Measures							
								Spasticity	Motor	Gait	Bal	ADL	ND	QoL	Safety
Zhang 2007 [14] ^#^	T: 35 (21/14) C: 35 (21/14)	T: 65.2 ± 11.2 C: 65.3 ± 11.4	T: 10.2 ± 5.3 d C: 10.2 ± 5.3 d	Limbs	T: AAPA + CI C: CM RH	2	14		FMA (U/L + L/L)			MBI			
Lu 2012 [15]	T: 25, C: 25, total: (27/23)	Total, range: 39–70	Total: 3 mo-3 yr	Ankle	T: AAPA + CI C: CM RH SA BA	4	24	TER, MAS	FMA (NR)						
Zhou 2013 [16]	T: 30 (17/13) C: 30 (15/15)	T: 59.76 ± 10.64 C: 58.65 ± 10.53	T: 32.76 ± 11.43 d C: 31.85 ± 10.71 d	Ankle	T: AAPA + CI C: CM RH	4	24	TER	FMA (L/L)						
Lu 2014 [17]	T: 60 (39/21) C: 60 (37/23)	T: 63.14 ± 5.31 C: 63.73 ± 5.75	T: 12.32 ± 3.51 d C: 12.87 ± 3.89 d	Ankle	T: AAPA + CI C: CM RH	8	48	TER	FMA (L/L)						
Xie 2017 [18]	T: 33 (20/13) C: 30 (21/9)	T: 62.8 ± 13.2 C: 65.5 ± 9.1	NR	Ankle	T: AAPA + CI C: CM RH	4	12	CSI	FMA (L/L)			MBI			
Zhang 2018 [19]	T: 30 (16/14) C: 30 (19/11)	T: 56.73 ± 14.98 C: 58.63 ± 15.98	T: 95.47 ± 47.59 d C: 90.90 ± 46.59 d	Hand	T: AAPA + CI C: CM RH BA	4	28	TER, MAS	FMA (H)						
Wang 2020 [20] ^#^	T: 33 (18/15) C: 34 (16/18)	T: 51 ± 9 C: 53 ± 10	T: 64.8 ± 38.2 d C: 61.9 ± 32.8 d	Hand	T: AAPA + CI C: CM RH	4	24	MAS	FMA (H + WR)			MBI			
Zheng 2020 [21]	T: 30 (18/12) C: 30 (17/13)	T: 55.39 ± 5.51 C: 55.41 ± 5.49	T: 5.11 ± 1.12 wk C: 5.24 ± 1.09 wk	Ankle	T: AAPA + CI C: CM RH HM	4	NR	TER	FMA (L/L)	GA					
Zhu 2020 [22]	T: 30 (13/17) C: 30 (16/14)	T: 63 ± 10 C: 64 ± 13	T: 19.9 ± 6.5 d C: 21.5 ± 5.2 d	U/L	T: AAPA + CI C: CM RH	4	20	TER, MAS	FMA (U/L)			MBI	NIHSS		
Wang 2021 [23] ^#^	T: 38 (25/13) C: 37 (28/9)	T: 56.89 ± 9.92 C: 59.32 ± 9.38	T: 50.74 ± 15.70 d C: 46.35 ± 15.53 d	U/L	T: AAPA + CI C: CM RH	2	12	MAS	FMA (NR)			MBI			AE
Zeng 2022 [24]	T: 55 (31/24) C: 55 (30/25)	T: 58.01 ± 4.53 C: 57.24 ± 4.65	NR	Limbs	T: AAPA + CI C: CM HM	8	40		FMA (U/L + L/L)		BBS				
Sang 2023 [25]	T: 42 (23/19) C: 42 (26/16)	T: 56.19 ± 9.78 C: 57.03 ± 9.77	NR	U/L	T: AAPA + CI C: CM RH	8	40	MAS	FMA (U/L)				NIHSS		
Wei 2024 [26] ^##^	T: 42 (25/17) C: 42 (24/18)	T: 60.80 ± 7.23 C: 61.22 ± 7.31	T: 50.65 ± 8.37 d C: 51.05 ± 8.52 d	Limbs	T: AAPA + CI C: CM RH EA	12	60	TER, MAS	FMA (U/L + L/L)	GA		MBI	NIHSS		AE
Wen 2024 [27]	T: 33 (23/10) C: 33 (21/12)	T: 60.47 ± 4.52 C: 59.38 ± 4.71	T: 2.27 ± 1.85 mo C: 2.13 ± 1.83 mo	Limbs	T: AAPA + CI C: CM RH	4	28	TER, MAS	FMA (L/L)		BBS				
Zhang 2024 [28]	T: 37 (24/13) C: 37 (25/12)	T: 59.73 ± 5.54 C: 59.41 ± 6.08	T: 38.90 ± 12.30 d C: 29.40 ± 13.30 d	U/L	T: AAPA + CI C: CM RH	4	28	TER, MAS	FMA (U/L)			MBI		SS-QoL	AE
Li 2026 [29]	T: 32 (28/4) C: 32 (27/5)	T: 53.9 ± 11.3 C: 55.8 ± 10.3	T: 104.6 ± 62.3 d C: 97.8 ± 54.9	L/L	T: AAPA + CI C: CM RH BA SET	4	20	MAS	FMA (L/L)	GA	BBS				AE

* Mean ± standard deviation. For ^#^ 3-arm or ^##^ 4-arm studies, only the arms relevant to the present comparison were extracted. AAPA, acupoint-to-acupoint penetrating acupuncture; ADL, activities of daily living; AE, adverse event; BA, body acupuncture; Bal, balance; BBS, Berg Balance Scale; C, control group; CI, control intervention; CM, conventional medical care; CSI, composite spasticity index; d, day; EA, electroacupuncture; F, female; FMA, Fugl-Meyer Assessment; GA, gait analysis; H, hand; HM, herbal medicine; L/L, lower limb; M, male; MAS, Modified Ashworth Scale; MBI, modified Barthel index; mo, month; ND, neurological deficit; NIHSS, National Institutes of Health Stroke Scale; NR, not reported; QoL, quality of life; RH, rehabilitation; SA, scalp acupuncture; SET, sling exercise therapy; SP, spasticity; SS-QoL, stroke-specific quality of life; T, treatment group; TER, total effective rate; TSS, time since stroke onset; Tx, treatment; U/L, upper limb; wk, week; WR, wrist; yr, years.

**Table 2:** Frequently used AAPA penetration routes and individual acupoints across included studies. (**A**) Frequently used AAPA penetration routes. (**B**) Frequently used individual acupoints for AAPA.

(**A**)			
**Rank**	**Acupoint Pair Used for AAPA**	**Anatomical Region**	**Frequency**
1	GB34 → SP9	L/L	9
2	LI4 → SI3	U/L	6
2	GB40 → KI6	L/L	6
4	LI11 → HT3	U/L	5
4	GB39 → SP6	L/L	5
6	LI15 → LU2	U/L	4
6	TE5 → PC6	U/L	4
8	SP6 → GB39	L/L	3
8	LR3 → KI1	L/L	3
8	LI4 → PC8	U/L	3
(**B**)			
**Rank**	**Individual Acupoint Used for AAPA**	**Anatomical Region**	**Frequency**
1	GB34	L/L	12
2	SP9	L/L	11
3	LI4	U/L	10
4	TE5	U/L	9
4	GB39	L/L	9
6	SP6	L/L	8
6	SI3	U/L	8
8	LI11	U/L	7
8	KI6	L/L	7
10	PC6	U/L	6
10	LI15	U/L	6
10	GB40	L/L	6

AAPA, acupoint-to-acupoint penetrating acupuncture; L/L, lower limb; U/L, upper limb. Note: Opposite penetration directions were treated as distinct acupoint pairs. In one study applying bidirectional penetration between LI4 and SI3, LI4 → SI3 and SI3 → LI4 were counted separately.

**Table 3:** Summary of findings and certainty of evidence assessed using GRADE.

Comparisons	Outcomes	Participants (Studies)	Risk of Bias	Inconsistency	Indirectness	Imprecision	Other Considerations	Overall Certainty of Evidence	Study Event Rates (%)		Anticipated Effects
									TG	CG	TG
Spasticity	MAS	600 (9 RCTs)	Very serious ^a^	Very serious ^b^	Not serious	Not serious	None	⨁◯◯◯ Very low ^a,b^	300	300	SMD 0.91 lower (1.23 lower to 0.59 lower))
Motor	FMA (motor)	264 (3 RCTs)	Very serious ^a^	Very serious ^b^	Not serious	Serious ^c^	None	⨁◯◯◯ Very low ^a,b,c^	132	132	MD 17.75 higher (10.29 higher to 25.20 higher)
	FMA (U/L)	218 (3 RCTs)	Very serious ^a^	Not serious	Not serious	Serious ^c^	None	⨁◯◯◯ Very low ^a,c^	109	109	MD 5.36 higher (3.31 higher to 7.42 higher)
	FMA (L/L)	433 (6 RCTs)	Very serious ^a^	Very serious ^b^	Not serious	Not serious	None	⨁◯◯◯ Very low ^a,b^	218	218	MD 3.89 higher (2.15 higher to 5.63 higher)
Gait	Gait speed	208 (3 RCTs)	Very serious ^a^	Not serious	Not serious	Serious ^c^	None	⨁◯◯◯ Very low ^a,c^	104	104	MD 6.49 higher (4.19 higher to 8.79 higher)
	Step length	144 (2 RCTs)	Very serious ^a^	Not serious	Not serious	Serious ^c^	None	⨁◯◯◯ Very low ^a,c^	72	72	MD 5.34 higher (1.74 higher to 8.93 higher)
	Cadence	208 (3 RCTs)	Very serious ^a^	Not serious	Not serious	Serious ^c^	None	⨁◯◯◯ Very low ^a,c^	104	104	MD 8.90 higher (5.70 higher to 12.11 higher
Balance	BBS	240 (3 RCTs)	Very serious ^a^	Not serious	Not serious	Serious ^c^	None	⨁◯◯◯ Very low ^a,c^	120	120	MD 6.76 higher (4.37 higher to 9.15 higher)
ADL	MBI	493 (7 RCTs)	Very serious ^a^	Very serious ^b^	Not serious	Not serious	None	⨁◯◯◯ Very low ^a,b^	248	245	MD 14.33 higher (8.58 higher to 20.12 higher)
Neurological deficit	NIHSS	228 (3 RCTs)	Very serious ^a^	Not serious	Serious ^d^	Serious ^c^	None	⨁◯◯◯ Very low ^a,c,d^	114	114	MD 1.75 lower (3.17 lower to 0.33 lower)

^a^. All or most included studies were judged to be at high risk of bias. ^b^. The Higgins’ I^2^ value exceeded 75% in the pooled analysis. ^c^. Number of participants was less than 400. ^d^. The NIHSS reflects overall neurological deficit rather than a spasticity-specific outcome.

## Data Availability

Data is contained within the article. Additional extracted data and analytic files are available from the corresponding author upon reasonable request.

## Abbreviations

The following abbreviations are used in this manuscript:

AAPA	Acupoint-to-acupoint penetrating acupuncture
ADL	Activities of daily living
AE	Adverse event
BBS	Berg Balance Scale
CI	Confidence interval
CNKI	China National Knowledge Infrastructure
CRIS	Clinical Research Information Service
FMA	Fugl–Meyer Assessment
GRADE	Grading of Recommendations Assessment, Development and Evaluation
ICTRP	International Clinical Trials Registry Platform
KISS	Korean Studies Information Service System
MAS	Modified Ashworth Scale
MBI	Modified Barthel Index
MCID	Minimal clinically important difference
MD	Mean difference
NIHSS	National Institutes of Health Stroke Scale
OASIS	Oriental Medicine Advanced Searching Integrated System
PRISMA	Preferred Reporting Items for Systematic Reviews and Meta-Analyses
PROSPERO	International Prospective Register of Systematic Reviews
QoL	Quality of life
RCT	Randomized controlled trial
RevMan	Review Manager software
RoB 2	Cochrane Risk of Bias 2
RR	Risk ratio
SMD	Standardized mean difference
SS-QoL	Stroke-specific quality of life
TER	Total effective rate

## Appendix A

**Table A1:** PRISMA 2020 Checklist.

Section and Topic	Item #	Checklist Item	Location Where Item Is Reported
TITLE			
Title	1	Identify the report as a systematic review.	Title
ABSTRACT			
Abstract	2	See the PRISMA 2020 for Abstracts checklist.	Abstract
INTRODUCTION			
Rationale	3	Describe the rationale for the review in the context of existing knowledge.	Section 1
Objectives	4	Provide an explicit statement of the objective(s) or question(s) the review addresses.	Section 1
METHODS			
Eligibility criteria	5	Specify the inclusion and exclusion criteria for the review and how studies were grouped for the syntheses.	Section 2.3 and Section 2.6
Information sources	6	Specify all databases, registers, websites, organizations, reference lists and other sources searched or consulted to identify studies. Specify the date when each source was last searched or consulted.	Section 2.2
Search strategy	7	Present the full search strategies for all databases, registers and websites, including any filters and limits used.	Section 2.2; Table A2
Selection process	8	Specify the methods used to decide whether a study met the inclusion criteria of the review, including how many reviewers screened each record and each report retrieved, whether they worked independently, and if applicable, details of automation tools used in the process.	Section 2.4
Data collection process	9	Specify the methods used to collect data from reports, including how many reviewers collected data from each report, whether they worked independently, any processes for obtaining or confirming data from study investigators, and if applicable, details of automation tools used in the process.	Section 2.4
Data items	10a	List and define all outcomes for which data were sought. Specify whether all results that were compatible with each outcome domain in each study were sought (e.g., for all measures, time points, analyses), and if not, the methods used to decide which results to collect.	Section 2.3.4 and Section 2.6
	10b	List and define all other variables for which data were sought (e.g., participant and intervention characteristics, funding sources). Describe any assumptions made about any missing or unclear information.	Section 2.4
Study risk of bias assessment	11	Specify the methods used to assess risk of bias in the included studies, including details of the tool(s) used, how many reviewers assessed each study and whether they worked independently, and if applicable, details of automation tools used in the process.	Section 2.5
Effect measures	12	Specify for each outcome the effect measure(s) (e.g., risk ratio, mean difference) used in the synthesis or presentation of results.	Section 2.6
Synthesis methods	13a	Describe the processes used to decide which studies were eligible for each synthesis (e.g., tabulating the study intervention characteristics and comparing against the planned groups for each synthesis (item #5)).	Section 2.3 and Section 2.6
	13b	Describe any methods required to prepare the data for presentation or synthesis, such as handling of missing summary statistics, or data conversions.	Section 2.6
	13c	Describe any methods used to tabulate or visually display results of individual studies and syntheses.	Section 2.6; Figure 2, Figure 3, Figure 4, Figure 5, Figure 6, Figure 7 and Figure 8; Table 1, Table 2 and Table 3; Figure A1; Table A4 and Table A5
	13d	Describe any methods used to synthesize results and provide a rationale for the choice(s). If meta-analysis was performed, describe the model(s), method(s) to identify the presence and extent of statistical heterogeneity, and software package(s) used.	Section 2.6
	13e	Describe any methods used to explore possible causes of heterogeneity among study results (e.g., subgroup analysis, meta-regression).	Section 2.6
	13f	Describe any sensitivity analyses conducted to assess robustness of the synthesized results.	Section 2.6
Reporting bias assessment	14	Describe any methods used to assess risk of bias due to missing results in a synthesis (arising from reporting biases).	Section 2.6
Certainty assessment	15	Describe any methods used to assess certainty (or confidence) in the body of evidence for an outcome.	Section 2.7
RESULTS			
Study selection	16a	Describe the results of the search and selection process, from the number of records identified in the search to the number of studies included in the review, ideally using a flow diagram.	Section 3.1; Figure 1
	16b	Cite studies that might appear to meet the inclusion criteria, but which were excluded, and explain why they were excluded.	Section 3.2; Figure 1; Table A3
Study characteristic	17	Cite each included study and present its characteristics.	Section 3.2; Table 1; Table A4
Risk of bias in studies	18	Present assessments of risk of bias for each included study.	Section 3.3; Figure 2
Results of individual studies	19	For all outcomes, present, for each study: (a) summary statistics for each group (where appropriate) and (b) an effect estimate and its precision (e.g., confidence/credible interval), ideally using structured tables or plots.	Section 3.4; Figure 3, Figure 4, Figure 5, Figure 6, Figure 7 and Figure 8; Figure A1
Results of synthesis	20a	For each synthesis, briefly summarize the characteristics and risk of bias among contributing studies.	Section 3.2, Section 3.3 and Section 3.4; Figure 2; Table 3
	20b	Present results of all statistical syntheses conducted. If meta-analysis was done, present for each the summary estimate and its precision (e.g., confidence/credible interval) and measures of statistical heterogeneity. If comparing groups, describe the direction of the effect.	Section 3.4; Figure 3, Figure 4, Figure 5, Figure 6, Figure 7 and Figure 8; Figure A1; Table 3
	20c	Present results of all investigations of possible causes of heterogeneity among study results.	Section 3.4
	20d	Present results of all sensitivity analyses conducted to assess the robustness of the synthesized results.	Section 3.5; Table A5
Reporting biases	21	Present assessments of risk of bias due to missing results (arising from reporting biases) for each synthesis assessed.	Section 3.5 (not assessed because fewer than 10 studies were included in each meta-analysis)
Certainty of evidence	22	Present assessments of certainty (or confidence) in the body of evidence for each outcome assessed.	Section 3.7; Table 3
DISCUSSION			
Discussion	23a	Provide a general interpretation of the results in the context of other evidence.	Section 4.1, Section 4.2, Section 4.3 and Section 4.4
	23b	Discuss any limitations of the evidence included in the review.	Section 4.5
	23c	Discuss any limitations of the review processes used.	Section 4.5
	23d	Discuss implications of the results for practice, policy, and future research.	Section 4.1, Section 4.4, Section 4.5 and Section 5
OTHER INFORMATION			
Registration	24a	Provide registration information for the review, including register name and registration number, or state that the review was not registered.	Section 2.1
	24b	Indicate where the review protocol can be accessed, or state that a protocol was not prepared.	Section 2.1 (PROSPERO registration record)
	24c	Describe and explain any amendments to information provided at registration or in the protocol.	Updated to reflect amendments made during the revision process.
Support	25	Describe sources of financial or non-financial support for the review, and the role of the funders or sponsors in the review.	Funding; Acknowledgments
Competing interests	26	Declare any competing interests of review authors.	Conflicts of Interest
Availability of data, code and other materials	27	Report which of the following are publicly available and where they can be found: template data collection forms; data extracted from included studies; data used for all analyses; analytic code; any other materials used in the review.	Data Availability Statement; Table 1, Table 2 and Table 3; Table A2, Table A3, Table A4 and Table A5; Figure 1, Figure 2, Figure 3, Figure 4, Figure 5, Figure 6, Figure 7 and Figure 8; Figure A1

**Table A2:** Complete search strategies for electronic databases and clinical trial registries.

Source Type	Source	Date Searched	Search Field	Search Terms	Filters/ Limits	Records Identified
Databases	PubMed	24 June 2026	Advanced	((((((((((((stroke[MeSH Terms]) OR (cerebrovascular disorders[MeSH Terms])) OR (cerebrovascular disorder[Title/Abstract])) OR (cerebrovascular disease[Title/Abstract])) OR (cerebrovascular accident[Title/Abstract])) OR (cerebral infarction[Title/Abstract])) OR (cerebral hemorrhage[Title/Abstract])) OR (ischemic stroke[Title/Abstract])) OR (hemorrhagic stroke[Title/Abstract])) OR (apoplexy[Title/Abstract])) OR (brain ischemia[Title/Abstract])) AND (((((((Muscle Spasticity[MeSH Terms]) OR (spasticity[Title/Abstract])) OR (spasm[Title/Abstract])) OR (spasticism[Title/Abstract])) OR (muscle hypertonia[Title/Abstract])) OR (muscle tightness[Title/Abstract])) OR (muscle stiffness[Title/Abstract]))) AND ((((((((((acupoint-to-acupoint needling[Title/Abstract]) OR (penetration acupuncture[Title/Abstract])) OR (penetration needle[Title/Abstract])) OR (penetration needling[Title/Abstract])) OR (penetrating acupuncture[Title/Abstract])) OR (penetrating needle[Title/Abstract])) OR (penetrating needling[Title/Abstract])) OR (Penetrative Acupuncture[Title/Abstract])) OR (Penetrative needle[Title/Abstract])) OR (Penetrative needling[Title/Abstract]))	None	5
	Embase			(‘cerebrovascular accident’/exp OR ‘cerebrovascular disease’/exp OR stroke OR cerebrovascular disorder OR cerebral infarction OR cerebral hemorrhage OR ischemic stroke OR hemorrhagic stroke OR apoplexy OR brain ischemia) AND (‘spasticity’/exp OR muscle spasticity OR spasm OR spasticism OR muscle hypertonia OR muscle tightness OR muscle stiffness) AND (acupoint-to-acupoint needling OR penetration acupuncture OR penetration needle OR penetration needling OR penetrating acupuncture OR penetrating needle OR penetrating needling OR Penetrative Acupuncture OR Penetrative needle OR Penetrative needling)		0
	Cochrane Library			[TiAbKw](stroke OR cerebrovascular disorder OR cerebrovascular disease OR cerebrovascular accident OR cerebral infarction OR cerebral hemorrhage OR ischemic stroke OR hemorrhagic stroke OR apoplexy OR brain ischemia) AND (spasticity OR muscle spasticity OR spasm OR spasticism OR muscle hypertonia OR muscle tightness OR muscle stiffness) AND (acupoint-to-acupoint needling OR penetration acupuncture OR penetration needle OR penetration needling OR penetrating acupuncture OR penetrating needle OR penetrating needling OR penetrative acupuncture OR penetrative needle OR penetrative needling)		13
	CNKI			[TKAb] (中风 OR 脑卒中 OR 脑梗死 OR 脑出血 OR 腦血管疾病 OR stroke OR cerebral infarction OR cerebral hemorrhage OR cerebrovascular disorder OR cerebrovascular accident) AND (透刺 OR 透针 OR penetration acupuncture OR penetration needling OR penetrating acupuncture OR penetrating needling OR penetrative acupuncture OR penetrative needling OR acupoint-to-acupoint needling) AND (痉挛 OR spasticity OR spasm OR spasticism OR muscle hypertonia OR muscle tightness OR muscle stiffness)		152
	Airiti Library			TKAb [ALL3] = {(中風 OR 中风 OR 腦卒中 OR 脑卒中 OR 腦中風 OR 脑中风 OR 腦梗死 OR 脑梗死 OR 腦梗塞 OR 脑梗塞 OR 腦出血 OR 脑出血 OR 腦血管疾病 OR 脑血管疾病 OR 腦血管意外 OR 脑血管意外 OR stroke OR “cerebral infarction” OR “cerebral hemorrhage” OR “ischemic stroke” OR “hemorrhagic stroke” OR “cerebrovascular disorder” OR “cerebrovascular disease” OR “cerebrovascular accident” OR apoplexy OR “brain ischemia”) AND (痙攣 OR 痉挛 OR 痙縮 OR 痉缩 OR 肌張力增高 OR 肌张力增高 OR 肌肉張力過高 OR 肌肉张力过高 OR 肌張力過高 OR 肌张力过高 OR 肌緊張 OR 肌紧张 OR 肌僵硬 OR spasticity OR spasm OR spasticism OR “muscle hypertonia” OR “muscle tightness” OR “muscle stiffness”) AND (透刺 OR 透針 OR 透针 OR 透穴刺 OR 透穴針刺 OR 透穴针刺 OR 透刺法 OR 透針法 OR 透针法 OR 穴位透刺 OR 穴位透針 OR 穴位透针 OR “acupoint-to-acupoint needling” OR “penetration acupuncture” OR “penetration needle” OR “penetration needling” OR “penetrating acupuncture” OR “penetrating needle” OR “penetrating needling” OR “penetrative acupuncture” OR “penetrative needle” OR “penetrative needling” OR “point-to-point needling” OR “through-needling” OR “through needling” OR “through acupuncture”)} OR {(痙攣 OR 痉挛 OR 痙縮 OR 痉缩 OR 肌張力增高 OR 肌张力增高 OR 肌肉張力過高 OR 肌肉张力过高 OR 肌張力過高 OR 肌张力过高 OR 肌緊張 OR 肌紧张 OR 肌僵硬 OR spasticity OR spasm OR spasticism OR “muscle hypertonia” OR “muscle tightness” OR “muscle stiffness”) AND (透刺 OR 透針 OR 透针 OR 透穴刺 OR 透穴針刺 OR 透穴针刺 OR 透刺法 OR 透針法 OR 透针法 OR 穴位透刺 OR 穴位透針 OR 穴位透针 OR “acupoint-to-acupoint needling” OR “penetration acupuncture” OR “penetration needle” OR “penetration needling” OR “penetrating acupuncture” OR “penetrating needle” OR “penetrating needling” OR “penetrative acupuncture” OR “penetrative needle” OR “penetrative needling” OR “point-to-point needling” OR “through-needling” OR “through needling” OR “through acupuncture”))} OR {(中風 OR 中风 OR 腦卒中 OR 脑卒中 OR 腦中風 OR 脑中风 OR 腦梗死 OR 脑梗死 OR 腦梗塞 OR 脑梗塞 OR 腦出血 OR 脑出血 OR 腦血管疾病 OR 脑血管疾病 OR 腦血管意外 OR 脑血管意外 OR stroke OR “cerebral infarction” OR “cerebral hemorrhage” OR “ischemic stroke” OR “hemorrhagic stroke” OR “cerebrovascular disorder” OR “cerebrovascular disease” OR “cerebrovascular accident” OR apoplexy OR “brain ischemia”) AND (痙攣 OR 痉挛 OR 痙縮 OR 痉缩 OR 肌張力增高 OR 肌张力增高 OR 肌肉張力過高 OR 肌肉张力过高 OR 肌張力過高 OR 肌张力过高 OR 肌緊張 OR 肌紧张 OR 肌僵硬 OR spasticity OR spasm OR spasticism OR “muscle hypertonia” OR “muscle tightness” OR “muscle stiffness”)}		35
	CiNii			{(stroke | “cerebrovascular disorders” | “cerebrovascular disorder” | “cerebrovascular disease” | “cerebrovascular accident” | “cerebral infarction” | “cerebral hemorrhage” | “ischemic stroke” | “hemorrhagic stroke” | apoplexy | “brain ischemia” | 脳卒中 | 脳血管障害 | 脳梗塞 | 脳出血 | 脳虚血) AND (spasticity | spasm | spasticism | “muscle hypertonia” | “muscle tightness” | “muscle stiffness” | 痙縮 | 痙攣 | 筋緊張亢進 | “筋肉のこわばり”) AND (“acupoint-to-acupoint needling” | “penetration acupuncture” | “penetration needle” | “penetration needling” | “penetrating acupuncture” | “penetrating needle” | “penetrating needling” | “Penetrative Acupuncture” | “Penetrative needle” | “Penetrative needling” | 透刺 | 透刺灸 | 透穴 | 透針)} OR { “acupoint-to-acupoint needling” | “penetration acupuncture” | “penetration needle” | “penetration needling” | “penetrating acupuncture” | “penetrating needle” | “penetrating needling” | “Penetrative Acupuncture” | “Penetrative needle” | “Penetrative needling” | 透刺 | 透刺灸 | 透穴 | 透針}		45
	ScienceOn			{(stroke | “cerebrovascular disorder” | “cerebrovascular accident” | “cerebrovascular disease” | “cerebral infarction” | “cerebral hemorrhage” | apoplexy | “brain ischemia” | “ischemic stroke” | “hemorrhagic stroke”) AND (“acupoint-to-acupoint needling” | “penetration acupuncture” | “penetration needle” | “penetration needling” | “penetrating acupuncture” | “penetrating needle” | “penetrating needling” | “penetrative acupuncture” | “penetrative needle” | “penetrative needling”) AND (spasticity | “muscle spasticity” | spasm | spasticism | “muscle hypertonia” | “muscle tightness” | “muscle stiffness”)} OR {(뇌졸중 | 뇌경색 | 뇌출혈 | 중풍 | 腦卒中 | 腦硬塞 | 腦出血 | 中風) AND (투자 | 透刺 | 透针) AND (경직 | 강직 | 硬直 | 强直)}		2
	KISS			{(stroke | “cerebrovascular disorder” | “cerebrovascular accident” | “cerebrovascular disease” | “cerebral infarction” | “cerebral hemorrhage” | apoplexy | “brain ischemia” | “ischemic stroke” | “hemorrhagic stroke”) AND (“acupoint-to-acupoint needling” | “penetration acupuncture” | “penetration needle” | “penetration needling” | “penetrating acupuncture” | “penetrating needle” | “penetrating needling” | “penetrative acupuncture” | “penetrative needle” | “penetrative needling”) AND (spasticity | “muscle spasticity” | spasm | spasticism | “muscle hypertonia” | “muscle tightness” | “muscle stiffness”)} OR {(뇌졸중 | 뇌경색 | 뇌출혈 | 중풍 | 腦卒中 | 腦硬塞 | 腦出血 | 中風) AND (투자 | 透刺 | 透针) AND (경직 | 강직 | 硬直 | 强直)}		0
	OASIS			{(stroke | “cerebrovascular disorder” | “cerebrovascular accident” | “cerebrovascular disease” | “cerebral infarction” | “cerebral hemorrhage” | apoplexy | “brain ischemia” | “ischemic stroke” | “hemorrhagic stroke”) AND (“acupoint-to-acupoint needling” | “penetration acupuncture” | “penetration needle” | “penetration needling” | “penetrating acupuncture” | “penetrating needle” | “penetrating needling” | “penetrative acupuncture” | “penetrative needle” | “penetrative needling”) AND (spasticity | “muscle spasticity” | spasm | spasticism | “muscle hypertonia” | “muscle tightness” | “muscle stiffness”)} OR {(stroke | “cerebrovascular disorder” | “cerebrovascular accident” | “cerebrovascular disease” | “cerebral infarction” | “cerebral hemorrhage” | apoplexy | “brain ischemia” | “ischemic stroke” | “hemorrhagic stroke”) AND (“acupoint-to-acupoint needling” | “penetration acupuncture” | “penetration needle” | “penetration needling” | “penetrating acupuncture” | “penetrating needle” | “penetrating needling” | “penetrative acupuncture” | “penetrative needle” | “penetrative needling”) AND (spasticity | “muscle spasticity” | spasm | spasticism | “muscle hypertonia” | “muscle tightness” | “muscle stiffness”)}		0
Clinical trial registries	ClinicalTrials.gov	21 June 2026	Basic	condition/disease: stroke AND other terms: spasticity AND intervention/treatment: acupuncture		9
	WHO ICTRP			title: stroke AND condition: spasticity AND intervention: acupuncture		6
	ChiCTR			(public title: stroke—target disease: spasticity—intervention: acupuncture) OR (public title: stroke—target disease: spasticity) OR (public title 脑卒中—target disease: 痉挛)		44
	jRCT		Free word	(search: stroke + name of the target: spasticity) OR (free word search: acupuncture + name of the target: stroke) OR (free word search: 脳卒中 + name of the target: 痙縮) OR (free word search鍼 + name of the target: 脳卒中)		11
	CRIS		Basic	(title: stroke AND condition: spasticity AND intervention: acupuncture) OR (title: 뇌졸중 AND condition: 경직 AND intervention: 침)		1

**Note: Non-English terms are original-language search terms corresponding to the English concepts of stroke, spasticity, and acupoint-to-acupoint penetrating acupuncture. They were retained to ensure reproducibility of the database searches**.

**Table A3:** Studies excluded after full-text review and reasons for exclusion.

Excluded Study	Reason for Exclusion
Xu Limin; Wang Sujuan (2018). Efficacy analysis of yin-yang meridian point-through-point acupuncture for spastic paralysis after stroke sequelae. Inner Mongolia Journal of Traditional Chinese Medicine, 37(1), 63.	Not RCT
Mi Jianping; Zhang Honglai; Fan Li; Meng Changrong (2004). Clinical observation of penetrating needle therapy for spasticity in post-stroke hemiplegia. Chinese Acupuncture & Moxibustion, 24(1), 11–12.	
Wu Changzheng; Ding Yi; Jiao Yang (2013). Scalp acupuncture plus body point-through-point acupuncture for post-stroke spastic paralysis: 42 cases. Guangming Journal of Chinese Medicine, 28(11), 2269, 2276.	
Li Wenjie; Liu Hongjian; Li Jintao (2023). Application effect of yin-yang balance point-through-point acupuncture combined with comprehensive rehabilitation training in patients with hemiplegic upper-limb spasticity. Clinical Research and Practice, 8(28), 98–101.	
Dang Yongsheng (2022). Efficacy of proprioceptive neuromuscular facilitation technique plus the Yin-Yang balance penetration acupuncture on patients with upper limb spastic hemiplegia after stroke and upper limb motor function and integral electromyography. Clinical Journal of Chinese Medicine, 14(16), 45–48.	
Pan Yongqing (2007). Clinical observation of point-through-point acupuncture plus electroacupuncture for increased muscle tone after stroke: 44 cases. Jiangsu Journal of Traditional Chinese Medicine, 39(1), 39–40.	
Feng DD, Fan JJ, Li P (2026). Rehabilitation effects of yin-yang balance penetrating acupuncture combined with contralaterally controlled functional electrical stimulation in patients with post-stroke upper-limb spastic hemiplegia. Chinese Journal of Convalescent Medicine, 35(7), 63–67.	
Yue Zenghui; Li Liang; Ye Yu; Jiang Jingming; Li Xiangrong (2010). Effect of acupuncture on serum amino acid levels in patients with post-stroke spastic paralysis. Journal of Hunan University of Chinese Medicine, 30(9), 212–215.	No spasticity-related outcome
Wang Ming (2011). The Clinical Research of Treating the Saccadic Paralysis of Stroke with Primarily Through Punctures. Master’s thesis, Hubei University of Chinese Medicine.	No limb acupoints used
Jiang Na; Cao Yi (2020). Clinical study of elongated-needle point-through-point acupuncture of the Governor Vessel for post-stroke dystonia. Journal of Anhui University of Chinese Medicine, 39(4), 65–68.	
Jia Zekun; Peng Yuanfeng; Jiang Na (2017). Clinical study of elongated-needle point-through-point acupuncture of the Governor Vessel for post-stroke spastic paralysis. Cardiovascular Disease Journal of Integrated Traditional Chinese and Western Medicine (Electronic), 5(30), 159, 162.	
Wang Xiaoyi (2017). Effect of Long-time Scalp Acupuncture Combined with Conventional Therapy on Limb Dysfunction in Young and Middle-aged Patients with Recovery-Phase Cerebral Hemorrhage. Master’s thesis, Zhejiang Chinese Medical University.	
Liu Jing; Bao Chunling; Zhang Guibo; Jiao Zhihua; Dong Guirong (2014). Effect of yin-yang regulating point-through-point acupuncture on reconstruction of walking function in patients with post-stroke spastic paralysis. Shanghai Journal of Acupuncture and Moxibustion, 33(1), 7–10.	
Li W, Zou WZ, Yin HB, Tong TT, Jiang LL, Sun DD, Zhou T (2026). The effect of elongated needle penetration of governor vessel acupoint groups on corticospinal tract remodeling in post-stroke patients with spastic hemiplegia. Journal of Anhui University of Chinese Medicine, 45(3), 60–65.	
Li Nana (2020). Observation of the efficacy of Huatan Tongluo Jiejing decoction combined with yin-yang meridian point-through-point acupuncture for post-stroke spastic paralysis. Journal of Practical Traditional Chinese Medicine, 36(2), 148–149.	Unreliable data
Chen Xiongjie; Gao Xuexia; Zhou Hongchun; Lai Ming; Wang Fang; Liu Xingxin (2025). Effect of point-toward-point acupuncture with elongated needles on lower-limb function in patients with post-stroke foot drop. Shanghai Journal of Acupuncture and Moxibustion, 44(6), 655–659.	AAPA vs. other interventions
Huang T. Wilarsini (2021). Clinical observation of five-hand-point point-through-point acupuncture for finger contracture in stroke sequelae. Master’s thesis, Liaoning University of Traditional Chinese Medicine.	
Wang Lei (2024). Observation on the clinical effect of through-acupuncture with awn needle on spastic paralysis caused by stroke. Master’s thesis, Anhui University of Chinese Medicine.	
An Chengfei (2022). Clinical observation on the treatment of foot inversion after stokr with “Tiaoheng Yinyang” penetrating acupuncture. Master’s thesis, Tianjin University of Traditional Chinese Medicine.	
Zhao Weifeng; Ren Yuanyuan; Zeng Baoxia; Lu He; Yue Yu; Zhang Ting; Zhou Zhijie (2021). Yin-yang penetration acupuncture with elongated needles for post-stroke spastic limb dysfunction: a randomized controlled trial. Chinese Acupuncture & Moxibustion, 41(7), 711–715.	
Zhang Nan (2020). Clinical observation on the treatment of handgrip after stroke with penetrating method. Master’s thesis, Tianjin University of Traditional Chinese Medicine.	
Zhang Yujia (2018). Regulatory effect of yin-yang meridians points penetration needling method for muscle tension of patients with stroke spastic paralysis. Master’s thesis, North China University of Science and Technology.	
Liu Daolong; Jiang Deyou; Chu Xuefei (2016). Observation of the efficacy of yin-yang meridian point-through-point acupuncture for spastic paralysis after stroke sequelae. Shaanxi Journal of Traditional Chinese Medicine, 37(8), 1068–1069.	
Su Meiluan (2015). Clinical study of penetration needling in antagonistic muscles in upper limb spastic paralysis after stroke. Master’s thesis, Guangzhou University of Chinese Medicine.	
Cheng Jijun; He Fen; Wang Yang; Zhang Fan (2015). Observation of the efficacy of penetrating acupuncture of antagonist muscles combined with rehabilitation training for post-stroke lower-limb spastic paralysis. Chinese Manipulation & Rehabilitation Medicine, 6(17), 25–26.	
Xing Chang (2015). Clinical observation of point-through-point acupuncture for spastic paralysis during recovery from ischemic stroke (liver-kidney deficiency pattern). Master’s thesis, Henan College of Chinese Medicine.	
Wang Shiwei (2015). Clinical study of flat-palm point-through-point acupuncture for hand spasm after ischemic stroke. Master’s thesis, Henan College of Chinese Medicine.	
Zhang Hongyan; Li Peifang (2013). Observation of the efficacy of antagonist-muscle point-through-point acupuncture for post-stroke spastic paralysis. Journal of Sichuan of Traditional Chinese Medicine, 31(9), 134–135.	
Xin Xin; Li Yanming; Wu Haoming; Zhuang Lixing (2012). Clinical study on optimized protocols of three acupuncture methods for spastic hemiplegia. Shaanxi Journal of Traditional Chinese Medicine, 33(1), 80–82.	
Jie Zihui (2011). Clinical observation of point-through-point acupuncture for spastic hemiplegia after stroke. Tianjin Journal of Traditional Chinese Medicine, 28(3), 199.	
Xing Xiaodong (2011). Clinical observation of finger muscular constricture after stroke with Hegu through Houxi acupuncture. Master’s thesis, Heilongjiang University of Chinese Medicine.	
Jiang Jingming; Yue Zenghui; Ye Yu; Li Liang; Li Xiangrong (2010). Clinical study of an acupuncture treatment protocol for relieving post-stroke spastic paralysis. Chinese Journal of Information on Traditional Chinese Medicine, 17(10), 14–16.	
Sun Shixiao; Wu Guijuan (2006). Clinical observation of stuck-needle plus point-through-point acupuncture for limb spasticity after stroke. Journal of Clinical Acupuncture and Moxibustion, 22(6), 37–38.	
Xu Yonghao; Wang Tao (1997). Observation of the efficacy of point-through-point acupuncture for post-stroke spastic hemiplegia: 40 cases. Journal of Ningxia Medical College, 19(2), 93–94.	
Luo Shuping; Zhu Cuilian; Chen Rui; Nie Bin (2025). Clinical study on point-penetration acupuncture mainly along the three yang channels of the hand for post-stroke finger spasm. Journal of New Chinese Medicine, 57(3), 100–104.	
Chen Qinda; Wang Shengguo (2025). Reflections on the immediate effect of acupoint point-through-point acupuncture for hand spasm after stroke. Contemporary Medical Symposium, 23(20), 116–119.	
Liu Xing; Deng Huiming (2018). Clinical effect of needle-knife therapy for elbow-joint spasm during stroke recovery. China Medical Herald, 15(21), 138–141.	
Zhang Qian; Wang Haidong (2022). Clinical observation of meridian-sinew theory combined with needle-knife therapy for elbow spasm during stroke recovery. Western Journal of Traditional Chinese Medicine, 35(7), 99–101.	
Yu Lu; Zhao Siyu; Feng Xugang (2017). Evaluation of elongated-needle point-through-point acupuncture supplemented with bloodletting for increased upper-limb muscle tone after stroke. Journal of China Prescription Drug, 15(1), 95–96.	AAPA combined with other interventions
Shao Chuxi (2016). Observation of the efficacy of five-point point-through-point acupuncture combined with Shixuan bloodletting for finger contracture after stroke. Master’s thesis, Liaoning University of Traditional Chinese Medicine.	
Li Fangfang (2023). Clinical observation of Qiao vessel point-through-point acupuncture combined with row needling of the Yangming meridian sinew for post-stroke strephenopodia. Master’s thesis, Tianjin University of Traditional Chinese Medicine.	
Zhang Junjie; Liu Yue; Li Kangju; Liu Xinjun (2020). Clinical observation on treating elbow spasm in stroke recovery by silver acupuncture. Clinical Journal of Chinese Medicine, 12(31), 56–59.	
Li Jixin (2015). Clinical observation of point-through-point acupuncture for post-stroke spastic hemiplegia. China’s Naturopathy, 23(9), 17–18.	
Wang Erzheng (2013). Observation on therapeutic effect of elongated needle therapy point-through-point Du meridian with biofeedback to treat spastic paralysis. Conference abstract/proceedings, 2013 Annual Meeting of the China Association of Acupuncture-Moxibustion/4th International Conference on Modernization of Traditional Chinese Medicine, Acupuncture Research and Internationalization Branch.	
Wang Erzheng; Xia Qing; Dong Yun; Xie Zongliang; Sun Peiyang (2009). Clinical study of elongated-needle point-through-point acupuncture of the Governor Vessel combined with bio-stimulation feedback for post-stroke spastic paralysis. Anhui Medical Journal, 30(12), 1397–1399.	
Gao Fei (2019). Baxie “burning mountain fire” technique combined with point-through-point acupuncture toward Laogong (PC8) for upper-limb spastic paralysis after ischemic stroke. Master’s thesis, Nanjing University of Chinese Medicine.	
Chen Lu (2023). Clinical study of antagonist-muscle point-through-point acupuncture for post-stroke lower-limb spastic paralysis. Master’s thesis, Guangzhou University of Chinese Medicine.	
Cao Lizhong; Liu Yue; Lu Yanqing; Yang Haitao; Su Meiluan (2014). Observation of the efficacy of point-through-point acupuncture of antagonist muscle groups for post-stroke spastic hemiplegia. Chinese Manipulation & Rehabilitation Medicine, 5(12), 49–51.	
Wang Kui; Liu Jingxing; Yang Yi; Xu Mingquan; Yang Qing (2013). Effect of point-through-point acupuncture combined with rehabilitation training on affected lower-limb function after stroke. Shanghai Journal of Acupuncture and Moxibustion, 32(7), 539–541.	
Shi Zhongmin (2010). The clinical study of using acupoint to acupoint penetrative needling in the treatment of spastic paralysis after stroke. Master’s thesis, Guangzhou University of Chinese Medicine.	
Liu Yue; Zhang Junjie; Kuang Weichuan; Xie Shuiping; Gao Haiyan; Zeng Kexue (2018). Clinical study of pair-acupoints penetration needling of antagonistic muscle for treatment of spastic paralysis of upper extremity after stroke. Yunnan Journal of Traditional Chinese Medicine and Materia Medica, 39(3), 55–57.	
Zhao Weiying (2020). Clinical observation of point-through-point acupuncture of antagonist muscles combined with electroacupuncture for post-stroke strephenopodia. Master’s thesis, Heilongjiang University of Chinese Medicine.	
Song Shuchang; Lu Zhi; Wang Runyun; Zhao Jianwen; Jia Lijuan (2014). Controlled study of electroacupuncture at Jiaji points combined with acupoint point-through-point acupuncture for post-stroke strephenopodia. Traditional Chinese Medicine Journal, 13(1), 48–50.	
Liu Yuheng (2019). Observation of the efficacy of point-through-point acupuncture combined with needle-knife therapy for post-stroke upper-limb spasticity. Electronic Journal of Clinical Medical Literature, 6(61), 53, 55.	
Yang Ziwen (2024). Efficacy of acupuncture through acupuncture combined with wax therapy in the treatment of foot varus after ischemic stroke: A randomized controlled trial. Master’s thesis, Tianjin University of Traditional Chinese Medicine.	
Wang Quanzhong; Chen Mingying; Zhang Yiling (2024). Cutaneous tapping plus acupoint point-through-point acupuncture combined with rehabilitation training for upper-limb elbow-flexion spasm after stroke: 30 cases. Fujian Journal of Traditional Chinese Medicine, 55(7), 48–50.	
Yang Ruiyong; Yan Luzhou; Zou Chaowen; Cheng Weifen (2013). Observation on therapeutic effect of point through point acupuncture add Jiawei Shaoyao Gancao Decoction of 35 post-stroke muscle spasm cases. Conference abstract/proceedings, 2013 Annual Meeting of the China Association of Acupuncture-Moxibustion/4th International Conference on Modernization of Traditional Chinese Medicine, Acupuncture Research and Internationalization Branch.	
Han Likun; Liu Yong; Li Chaosheng; Di Meiqi; Ni Chenfei; Liu Min; Sui Chenyan (2024). Effects of Xuefu Zhuyu decoction combined with yin-yang regulating point-through-point acupuncture on hemorheological indicators and TCM syndrome scores in patients with stroke sequelae. Journal of Chronic Diseases, 25(8), 1121–1125.	
Yu Chuan; Wang Linpeng (2014). Observation of the efficacy of twelve point-through-point acupuncture methods combined with fire needling for increased muscle tone after ischemic stroke. Journal of Clinical Acupuncture and Moxibustion, 30(12), 51–53.	
Li Qiujia (2022). Clinical observation of meridian point-through-point acupuncture combined with grain-sized moxibustion for upper-limb spasticity after stroke. Journal of Guangxi University of Chinese Medicine, 25(3), 13–16.	
Zhou Han; Hu Xiaohong (2016). Clinical study of acupuncture combined with rehabilitation training for spastic strephenopodia in stroke patients. Asia-Pacific Traditional Medicine, 12(24), 123–124.	
Li Ji; Long Hulin; Li Dehua (2016). Clinical observation of acupuncture combined with rehabilitation training for spastic strephenopodia in stroke patients. Sichuan Medical Journal, 37(3), 332–334.	
Wu Wei (2011). Observation of the efficacy of acupuncture combined with rehabilitation training for strephenopodia on the hemiplegic side after stroke. Shanghai Journal of Acupuncture and Moxibustion, 30(5), 321–323.	
Wang Na (2019). Clinical analysis of acupuncture combined with rehabilitation training for spastic strephenopodia in stroke patients. Electronic Journal of Clinical Medical Literature, 6(48), 44–46.	
Wang Ying (2012). Observation of the efficacy of acupuncture combined with exercise therapy for post-stroke strephenopodia: 60 cases. Hebei Journal of Traditional Chinese Medicine, 34(3), 412–413.	
Zhou Yaran (2011). Combined penetrating needling and kinesiotherapy for treating post-stroke strephenopodia. Shanghai Journal of Acupuncture and Moxibustion, 30(5), 324–326.	
Jiang Xue; Ye Yuqin; Xu Ping (2015). Clinical observation through acupuncture and rehabilitation therapy on strephenopodia after stroke. World Journal of Integrated Traditional and Western Medicine, 10(10), 1391–1393.	
Chen Tianlong (2016). The clinical study of acupoint penetration needling with meridian of Foot-Tai YANG through Yingqiao meridian in patients with strephenopoida after stroke. Master’s thesis, Guangzhou University of Chinese Medicine.	AAPA vs. AAPA with different protocols
Wang YY (2026). Therapeutic effects of mang needle yin-yang penetration needling method combined with Yiqi Huayu Decoction on post-stroke limb spasticity and its impact on apoptosis-related indicators. Heilongjiang Medical Journal, 50(5), 560–562.	
Zhang T, Shi WY, Zhuo CH, Yan S, Duan QY, Tu L, Wang Z (2025). Application effects of wrist-ankle acupuncture combined with yin-yang balance penetrating acupuncture in patients with post-stroke upper-limb spastic hemiplegia. Chinese Journal for Clinicians, 53(10),1239–1242.	
Li Zhe; Lü Jibao (2019). Observation of the efficacy of point-through-point acupuncture with round-sharp needle for spastic paralysis after apoplexy. Guangxi Medical Journal, 41(20), 2644–2647.	No AAPA applied
Jiao Zhihua; Dong Guirong (2009). Regulate effects of Yin-Yang balancing penetration acupuncture on treating ischemic patients with muscle tension: A randomized controlled clinical observation. Conference abstract, Proceedings of the 2009 Annual conference of the China Association of acupuncture-moxibustion.	Abstract (results not reported)
Wang Yuan; Dong Guirong (2009). Clinical observation on effect of Yin-Yang balancing penetration acupuncture therapy in the treatment of hemorrhage apoplexy with muscular tension dysfunction. Conference abstract, Proceedings of the 2009 Annual conference of the China Association of acupuncture-moxibustion.	

**Table A4:** Details of the acupoint-to-acupoint penetrating acupuncture in the included studies.

Study ID	Penetration Route (Acupoints of Insertion → Target)	Needle Specifications (Diameter × Length, mm)	Insertion Depth (mm)	Insertion Angle	Manual Stimulation Techniques	Needle Retention Time (min)
Zhang 2007 [14]	LI15 → LU2; LI11 → HT3; TE5 → PC6; LI4 → SI3; GB30 → BL54; ST34 → SP10; ST32 → SP13; SP9 → GB34; SP6 → GB39; LR3 → KI1	Filiform needle, length 75–100 ^§^	NR	NR	Lifting–thrusting, twirling	30
Lu 2012 [15]	^a^ SP3 → BL65; ^b^ GB40 → KI6; ^c^ KI8 → BL59; ^d^ GB34 → SP9	NR	^a,c^ 62.5–75, ^b^ 75–100, ^d^ 50–62.5^§^	^a^ Subcutaneous (10°), ^b,c^ oblique (45°), ^d^ perpendicular (90°)	Rapid twirling	30
Zhou 2013 [16]	^d^ GB34 → SP9; ^b^ GB40 → KI6; ^e^ SP6 → GB39	Filiform needle, ^d,e^ 0.30 × 40 or ^b^ 0.30 × 25	^d^ 20–25, ^e^ 12.5–20 ^§^	^b^ Oblique (45°), ^e^ perpendicular (90°)	Lifting-thrusting, rapid twirling	30
Lu 2014 [17]	GB34 → SP9; BL60 → KI3; BL62 → KI6	Filiform needle, 0.30 × 60	37.5–50 ^§^	NR	Twirling	30
Xie 2017 [18]	GB34 → SP9; GB39 → SP6; GB40 ^b^ → KI6; BL60 → SI3	Filiform needle, 0.25 × 40	NR	^b^ Oblique (45°); others perpendicular (90°)	Twirling	^b^ 0; others 30
Zhang 2018 [19]	LI4 ↔ SI3 (bidirectional)	Filiform needle, 0.25 × 40 or 80	30–37.5 ^§^	NR	Lifting–thrusting, twirling, sparrow-pecking	30
Wang 2020 [20]	^f^ LI4 → SI3; ^g^ TE5 → TE6	Filiform needle, 0.30 × 40	^f^ 20–30, ^g^ 25–30	Subcutaneous (15°)	Lifting–thrusting, twirling	30
Zheng 2020 [21]	^d^ GB34 → SP9; ^b^ GB40 → KI6; GB39 → SP6	Filiform needle, 0.35 × 75	^d^ 70–75; others 60–70	^d^ Perpendicular (90°), ^b^ oblique (45°)	NR	30
Zhu 2020 [22]	LI15 → LU2; LI11 → HT3; TE5 → PC6; LI4 → PC8; ^d^ GB34 → SP9; GB39 → SP6; BL60 → KI3; LR3 → KI1	Silver needle, 0.30 × 40	^d^ 50–60; others 35–38	NR	Lifting–thrusting	20
Wang 2021 [23]	^h^ LI15 → LU2; ^i^ TE13 → TE10; ^j^ TE6 → TE5; ^k^ LI5 → LI6	Filiform needle, 0.30 × 25~70	^h^ 50–70; ^i,j^ 40–50; ^k^ 25–30	NR	NR	30
Zeng 2022 [24]	* LI15 → LI11; * LI11 → LI7; ^#^ TE5 → PC6; ^#^ LI4 → PC8; * GB30 → GB31; ^#^ GB34 → SP9; * GB34 → GB39; ^#^ GB39 → SP6; ^#^ GB40 → KI6	Long needle, * 0.60 × 100 for deep muscle layer or ^#^ 0.40 × 50 for superficial layer	NR	NR	Lifting–thrusting	30
Sang 2023 [25]	LI15 → HT1; PC6 → TE5; LI11 → HT3; LI4 → PC8	NR, 0.30 × 40	35–38	NR	NR	20
Wei 2024 [26]	TE5 → PC6; LI4 → SI3; LI11 → HT3; GB39 → SP6; SP9 → GB34; GB30 → GB31	NR, Length 75 ^§^	NR	NR	NR	20
Wen 2024 [27]	^†^ LI15 → LU2; ^†^ LI11 → HT3; ^†^ TE5 → PC6; ^†^ LI4 → PC8; ^‡^ GB34 → SP9; ^‡^ GB39 → SP6; ^‡^ BL60 → KI3; ^‡^ LR3 → KI1	Silver needle, ^†^ 0.30 × 40 or ^‡^ 75	^†^ 50–60, ^‡^ 35–38	Oblique (45°)	NR	20
Zhang 2024 [28]	^l^ PC2 → LU5; ^l^ EX-UE (mid-upper arm) → TE5; ^f^ LI4 → SI3	Long needle, 0.35 × 100	^l^ 50–75, ^f^ 40–60	NR	NR	30
Li 2026 [29]	LI8 → GB3; GB34 → SP9, ^†^ BL59 → EX-HN5, ^d^ GB40 → KI6	Filiform needle, 0.30 × ^†^ 40 or 75	^†^ 20–40, ^d^ 40–60, 60–70	Perpendicular	Lifting–thrusting, twirling	30

Note: Lowercase letters (^a–l^) and symbols ^(*, #, †, ‡^, where used) are row-specific linkage markers. Within the same study row, matching markers indicate that the corresponding AAPA route and technical parameters, such as needle size, insertion depth, insertion angle, or target tissue layer, belong together. These markers have no meaning across different study rows and do not indicate statistical significance. ^§^ Values originally reported in cun (寸) were converted to millimeters (1 cun = 25 mm).

**Table A5:** Sensitivity analyses of primary outcome (modified Ashworth Scale).

Study Excluded	Pooled Standardized Mean Difference	95% Confidence Interval
Lu 2012 [15]	−0.75	−0.94 to −0.56
Zhang 2018 [19]	−0.92	−1.27 to −0.56
Wang 2020 [20]	−0.92	−1.28 to −0.56
Zhu 2020 [22]	−0.92	−1.28 to −0.56
Wang 2021 [23]	−0.95	−1.31 to −0.59
Sang 2023 [25]	−0.98	−1.32 to −0.63
Wen 2024 [27]	−0.95	−1.31 to −0.59
Zhang 2024 [28]	−0.96	−1.32 to −0.61
Li 2026 [29]	−0.86	−1.19 to −0.52
